# Leveraging social media to mitigate HPV vaccine service disruptions in Abuja, Nigeria

**DOI:** 10.1371/journal.pone.0352986

**Published:** 2026-07-09

**Authors:** Sohail Agha, Khadija Hassan, T. O. Olusanya, Hauwa Abbas, Drew Bernard, Ifeanyi Nsofor

**Affiliations:** 1 Department of Epidemiology, University of Washington, Seattle, United States of America; 2 Behavioral Insights Lab, Seattle, United States of America; 3 Silver Lining for the Needy Initiative, Abuja, Nigeria; 4 WellaHealth, Abuja, Nigeria; 5 Upswell, Seattle, United States of America; 6 Centre for Family Health Initiative, Abuja, Nigeria; VITAL Pakistan Trust, PAKISTAN

## Abstract

**Introduction:**

Market priming strategies use communication to prepare target audiences for service uptake when services become available. In 2025, a health worker strike in Abuja, Nigeria resulted in the suspension of HPV vaccination services for approximately four months. Services were temporarily restored for five days (July 16–20, 2025) during a Maternal, Newborn, and Child Health (MNCH) week. This study assessed whether caregiver recall of a digital market priming campaign was associated with HPV vaccine uptake during this brief service resumption.

**Methods:**

We conducted a cross-sectional exit survey of 271 caregivers of adolescent girls aged 9–17 years attending vaccination sites during MNCH week. The survey collected data on socio-demographic characteristics, Facebook use, recall of HPV vaccine advertising on Facebook or Instagram, and caregiver-reported adolescent vaccination status. Survey findings were triangulated with administrative data on the number of HPV vaccinations delivered during the MNCH week. Analyses included descriptive statistics and multivariable logistic regression. A two-stage residual inclusion (2SRI) model was used as a sensitivity analysis to assess potential bias from unobserved confounding related to social media use. Ward-level regression analyses using administrative data compared vaccinations delivered in wards targeted by the digital campaign with those in non-targeted wards.

**Results:**

Overall, 33.6% of caregivers recalled HPV vaccine advertising on Facebook or Instagram, and 64.2% reported that their adolescent had received the HPV vaccine. Message recall was associated with higher odds of caregiver-reported vaccination (adjusted odds ratio = 3.36; 95% CI: 2.75–4.11). Model-predicted marginal effects indicated a 23.3 percentage-point difference in reported vaccination probability between caregivers who did and did not recall messages, corresponding to an estimated 7.9 percentage-point difference in vaccination prevalence when applied to the caregiver population attending vaccination services. The 2SRI analysis found no evidence of endogeneity (p = 0.736). Administrative data were consistent with survey findings, suggesting an approximately 3 percentage-point higher vaccination coverage in wards targeted by the digital campaign compared with control wards during MNCH week.

**Conclusions:**

Caregiver recall of a digital market priming campaign was associated with higher HPV vaccination uptake during a short-term resumption of services following a prolonged disruption. These findings suggest that insights-informed, subnational digital priming strategies—when combined with complementary offline approaches to ensure equitable reach—may help maintain caregiver readiness and support HPV vaccine delivery during periods of service instability.

## Introduction

Human papillomavirus (HPV) is a leading cause of cervical cancer, which disproportionately affects women in low- and middle-income countries (LMICs). Despite the high efficacy of HPV vaccines and the World Health Organization’s global elimination targets [1,2], vaccination coverage in many LMICs remains well below the 90% goal. Vaccine rollouts in these settings frequently face challenges related to supply constraints, service delivery disruptions, and caregiver hesitancy. Together, these challenges highlight the importance of communication strategies that shape demand and help ensure that caregivers are prepared to accept HPV vaccination when services become available.

Market priming strategies—defined here as communication activities conducted before or during vaccine introduction or service changes to prepare target audiences for uptake—are increasingly used in HPV vaccination programs. Recent national examples include Nigeria’s 2023 pre-introduction campaign emphasizing cervical cancer burden and eligibility [3], Kenya’s 2023–2024 “one dose protects” campaign supporting the transition from a two-dose to a single-dose schedule [4], and Malawi’s communication efforts to build confidence in HPV vaccination [5]. These initiatives illustrate how demand-shaping communication can support vaccine adoption, particularly in settings where services are newly introduced or undergoing change. Conversely, ineffective or poorly timed communication has been shown to undermine HPV vaccine uptake [6].

Evidence on interventions that increase HPV vaccine uptake in LMICs remains limited. While a range of communication and educational strategies have been implemented, including school-based and community-based campaigns, systematic reviews highlight substantial gaps in rigorous evaluations, particularly in LMIC contexts [7,8].

Evidence from mass media health communication interventions across a broad range of health behaviors, including vaccination, suggests that such campaigns are often associated with modest but meaningful changes in behavior, frequently in the range of several percentage points [9–11]. These estimates provide contextual reference points from prior studies rather than benchmarks or expected effects for specific interventions.

However, little is known about the role of market priming strategies at the subnational level, particularly during periods when vaccine services are disrupted or intermittently available. In addition, evidence on the effects of digital communication interventions in LMICs remains limited [12]. Understanding whether digital demand-shaping efforts are associated with caregiver readiness to vaccinate during service interruptions is an important gap for immunization programs in these settings.

This study examines a digital media–based market priming strategy implemented in the Federal Capital Territory (FCT) of Abuja, Nigeria, during a period of service disruption. In 2025, a health worker strike resulted in the suspension of public HPV vaccination services for approximately four months, after which services were temporarily restored for five days (July 16–20, 2025) during a Maternal, Newborn, and Child Health (MNCH) week. The digital campaign was implemented in anticipation of this brief service resumption and was designed to maintain caregiver readiness by supporting both ability (e.g., information on service availability) and motivation through behavioral insights–informed messaging [13].

The campaign was informed by the Fogg Behavior Model, which conceptualizes behavior as occurring when sufficient motivation and ability coincide with an appropriate prompt [14]. In this study, the model informed message design, while message recall and vaccination status served as observable indicators related to these constructs rather than direct measures of motivation or ability. This study assessed whether caregiver recall of a digital market priming campaign was associated with HPV vaccine uptake during a brief resumption of services in Abuja, Nigeria.

## Methods

### Study design and context

We conducted an observational assessment combining a caregiver exit survey with aggregate service delivery data from administrative records. The study took place during Abuja’s Maternal, Newborn, and Child Health (MNCH) week, when public HPV vaccination services were temporarily restored for five days (July 16–20, 2025) following an approximately four-month health worker strike earlier in 2025. The assessment examined the association between caregiver recall of HPV vaccine messages delivered via social media and caregiver-reported adolescent HPV vaccination status during this brief service resumption.

Prior to the digital priming campaign assessed in this study, a behavioral insights survey informed by the Fogg Behavior Model was conducted in August 2024 to examine factors related to caregivers’ motivation and ability to vaccinate their children against HPV [15]. Findings from this formative research informed the development and refinement of social media messaging implemented between September and November 2024 in Abuja, Nasarawa, and Adamawa states. Visual assets developed during this earlier phase were subsequently reused and adapted for the July 2025 digital priming campaign, with updated framing and calls to action reflecting the MNCH-week service resumption. The present analysis focuses exclusively on data collected during the July 2025 MNCH week and does not include data from these earlier activities.

### Survey population and sampling

The exit survey targeted caregivers of adolescent girls aged 9–17 years in three of the six area councils of the Federal Capital Territory (Bwari, Kuje, and Abuja Municipal Area Council [AMAC]), where the digital priming campaign had been implemented. Data collection occurred at public primary health centres (PHCs), outreach vaccination events, and selected private pharmacies during MNCH week.

Within each data collection site, interviewers used systematic sampling, approaching every second eligible caregiver. Eligibility was determined by confirming that the respondent was a caregiver of a girl aged 9–17 years who had attended the facility or pharmacy during the MNCH week. Field supervisors monitored interviewer adherence to sampling procedures through spot checks and real-time review of submitted survey forms.

Caregivers recruited through private pharmacies were approached following service use. These pharmacies were purposively selected based on their participation in a WellaHealth-supported pharmacist training initiative focused on HPV vaccination counseling. Pharmacists first obtained permission for the study team to contact the caregiver, after which trained interviewers conducted telephone interviews. Interviewer time was allocated proportionally across PHCs, outreach events, and pharmacies to reflect client flow and service volume.

We aimed to complete 300 caregiver exit interviews, which would provide approximately ±6 percentage-point precision around an estimated vaccination proportion of 50%, accounting for clustering by recruitment site using a design effect of 1.5. Due to the short duration of HPV vaccination service availability during MNCH week, the final achieved sample included 271 caregivers.

### Data collection

Caregivers at PHCs, outreach events, and pharmacies were interviewed using a structured, close-ended, interviewer-administered questionnaire. The instrument collected data on:

- caregiver socio-demographic characteristics (age, gender, education);
- mobile phone ownership and frequency of Facebook use;
- caregiver recall of HPV vaccination messages on Facebook or Instagram in the preceding three months; and
- caregiver-reported HPV vaccination status of the adolescent girl.

Advertisements were delivered jointly across Facebook and Instagram using Meta’s advertising platform; platform-specific exposure could not be distinguished and was therefore not measured separately.

The questionnaire was pretested prior to implementation. Data collectors received standardized training and conducted practice interviews before starting fieldwork. Interviews were administered using tablet devices, with responses entered directly into a secure electronic data capture platform. Field supervisors monitored interviews in real time to ensure adherence to study protocols and data quality. Survey data were collected between July 16 and July 20, 2025.

### Ethical considerations

The study protocol, including the consent process, was reviewed and approved by the National Health Research Ethics Committee of Nigeria (NHREC) (protocol number NHREC/01/01/2007–17/08/2024; approval number NHREC/01/01/2007–29/08/2024), approved on 29 August 2024 and valid through 28 August 2025 [16]. Informed consent was obtained from all participants prior to data collection. Written informed consent was obtained for face-to-face interviews conducted at public primary health centers and outreach events. Informed verbal consent was obtained for telephone interviews, in accordance with ethics committee–approved procedures, and was documented by trained interviewers prior to survey administration. All participants were caregivers aged 18 years or older, and no personally identifiable information was collected. Additional information regarding the ethical, cultural, and scientific considerations specific to inclusivity in global research is included in the Supporting Information (S1 Checklist). The completed PLOS human subjects research checklist is provided in the Supporting Information (S2 Checklist).

### Message content and engagement strategy

The digital market priming campaign employed multiple short-form social media advertisements informed by behavioral insights research and iterative testing conducted earlier. Advertisements featured locally relevant themes and trusted messengers, including community pharmacists, and were designed to address common caregiver concerns about HPV vaccination, such as safety, cost, eligibility, and fertility-related misconceptions.

Several visual assets and message images used in the digital priming campaign were developed during earlier behavioral insights–informed campaign activities conducted in late 2024. These assets were reused during the 2025 priming campaign to ensure message continuity and efficiency, while accompanying text and calls to action were adapted to reflect the timing, service availability, and objectives of the MNCH-week vaccination opportunity. Thus, while the images were not newly created for the priming phase, their deployment and framing were specific to the service resumption period assessed in this study.

In response to service disruptions and uncertainty about vaccine availability, a subset of advertisements encouraged caregivers to sign up to receive notifications when HPV vaccination services became available in their area. This approach allowed the campaign to maintain engagement during periods of service interruption and enabled follow-up messaging when vaccination opportunities arose. Messages consistently emphasized that HPV vaccination was free, time-limited, and available for girls aged 9–14 years, and provided information on where services could be accessed during MNCH week.

During the campaign period, social media advertisements were delivered weekly to approximately 150,000 caregivers across targeted wards. A total of 30 distinct advertisement ‘creatives’—defined as unique combinations of images, text, and calls to action—were rotated during the campaign, each reaching an average of approximately 130,000 caregivers per week. Differences between these reach estimates reflect overlap in audience exposure across creatives, variation in delivery across Facebook and Instagram through Meta’s advertising platform, and platform-level reporting metrics, rather than distinct or mutually exclusive audiences. As a result, cumulative reach over the campaign period exceeds weekly reach but does not represent unique individuals.

Multiple creatives were rotated and adapted over time to improve relevance and engagement while maintaining consistent core themes related to motivation, trust, and caregivers’ ability to act. Caregiver recall of these messages was later assessed in the exit survey and used as the primary exposure variable in individual-level analyses.

### Measures

The primary outcome was caregiver-reported HPV vaccination status of the adolescent girl. The key independent variable was caregiver recall of HPV vaccine messaging delivered via Facebook or Instagram. Caregiver socio-demographic characteristics (age, gender, education) were included as control variables. Frequency of Facebook use (never, infrequent, once per day, multiple times per day) was measured as a categorical variable and was used as an instrument in a two-stage residual inclusion (2SRI) analysis to assess potential endogeneity of message recall [17].

### Statistical analysis

Survey analyses proceeded in three stages. First, descriptive statistics summarized sample characteristics. Second, bivariate analyses examined associations between message recall and vaccination status. Third, multivariable logistic regression models estimated the association between message recall and caregiver-reported vaccination status, adjusting for caregiver socio-demographic characteristics. Because caregivers who are more health-engaged may be both more likely to recall social media messages and more likely to vaccinate their children, we conducted an instrumental variable analysis as a **sensitivity check** to assess potential endogeneity of message recall. Frequency of Facebook use was used to predict recall of HPV vaccine messages in the first stage. The resulting residuals were included in the second-stage vaccination model using a two-stage residual inclusion (2SRI) approach, which is appropriate for models with a binary outcome variable.

Multicollinearity among covariates was assessed using variance inflation factors, and model fit was evaluated using standard goodness-of-fit statistics. All hypothesis tests were two-tailed, with statistical significance assessed at **α = 0.05**. No missing data were observed for variables included in the analyses.

Separately, multivariable regression models adjusting for ward population were used to examine differences in the number of HPV vaccinations delivered across wards. Regression analyses accounted for clustering by recruitment location (PHC, outreach event, pharmacy) to obtain robust standard errors [17]. All analyses were conducted using Stata [17].

### Service delivery data and intervention vs control wards

Aggregate administrative data on the number of HPV vaccinations provided during the July 16–20, 2025 MNCH week were obtained from local government health authorities.

In two area councils—the Abuja Municipal Area Council (AMAC, population approximately 4.2 million) and Kuje Area Council (population approximately 527,473)—wards were classified as intervention or control areas based on whether geofenced social media advertisements promoting HPV vaccination were delivered to caregivers residing in those wards. Assignment to intervention or control status was non-random and based on programmatic considerations. Not all caregivers residing in intervention wards were necessarily exposed to or recalled the digital messages. Ward-level comparisons therefore reflect differences in vaccination uptake in areas targeted by the digital campaign, rather than individual-level exposure effects.

Ward-level denominators for girls aged 9–14 years were estimated using ward population projections from local government sources combined with age-specific population proportions from the United Nations World Population Prospects [18]. These estimates are subject to uncertainty associated with population projections. Denominators were estimated at 54,831 and 6,857 for the AMAC and Kuje area councils, respectively. The number of HPV vaccinations provided during MNCH week was compared between intervention and control wards to assess differences in vaccination delivery associated with ward-level targeting, recognizing the non-randomized nature of the comparison.

## Results

### Sample characteristics

A total of 271 caregivers of adolescent girls participated in the exit survey (Table 1, Column 1). Most respondents were aged 30–39 years (55%), and the majority were women (85.6%). Approximately 5.9% had no formal education, 24.7% had completed SSE/GCE, and 35.4% had attained a higher national diploma (HND) or higher education. Facebook use was common, with 58.3% of caregivers reporting use multiple times per day and an additional 19.2% reporting daily use. Overall, 33.6% of caregivers recalled seeing HPV vaccine–related messages on Facebook or Instagram in the preceding three months, and 64.2% reported that their adolescent girl had received the HPV vaccine. Vaccination status reported in the exit survey reflects caregivers attending vaccination sites during MNCH week and should not be interpreted as population-level coverage.

**Table 1:** Caregiver characteristics and their relationship with HPV vaccination.

	Caregiver characteristics	Caregiver saw information on the HPV vaccine		Caregiver reported that adolescent was vaccinated	
Variables	(Column 1) n (%)	(Column 2) n (%)	P value	(Column 3) n (%)	P value
**Age of caregiver**			0.455		0.086
18-29	34 (12.5)	12 (35.3)		18 (52.9)	
30-39	149 (55.0)	54 (36.2)		104 (69.8)	
40 and older	88 (32.5)	25 (28.4)		52 (59.1)	
**Gender of caregiver**			0.688		0.707
Male	39 (14.4)	12 (30.8)		24 (61.5)	
Female	232 (85.6)	79 (34.1)		150 (64.7)	
**Education of caregiver**			0.062		<0.001
No formal education	16 (5.9)	2 (12.5)		6 (37.5)	
Primary School Certificate	18 (6.6)	2 (11.1)		8 (44.4)	
SSE/GCE	67 (24.7)	23 (34.3)		34 (50.7)	
Ordinary National Diploma (OND)	73 (26.9)	26 (35.6)		52 (71.2)	
Higher National Diploma (HND) or more	96 (35.4)	38 (39.6)		74 (77.1)	
**Frequency of use of Facebook**			<0.001		0.004
Never	34 (12.5)	3 (8.8)		17 (50.0)	
Infrequent	28 (10.0)	4 (14.8)		11 (40.7)	
Once a day	52 (19.2)	16 (30.8)		33 (63.5)	
Multiple times a day	158 (58.3)	68 (43.0)		113 (71.5)	
**Caregiver saw information on the HPV vaccine in the last 3 months**					<0.001
No	180 (66.4)	–		99 (55.0)	
Yes	91 (33.6)	–		75 (82.4)	
**Adolescent vaccinated**					
No	97 (35.8)	–		–	
Yes	174 (64.2)	–		–	
Total	271 (100)	91 (33.6)		174 (64.2)	

### Bivariate associations

Message recall (Table 1, Column 2) did not vary significantly by caregiver age, gender, or education. Recall was, however, associated with frequency of Facebook use: 43.0% of caregivers who reported using Facebook multiple times per day recalled HPV vaccine messages, compared with 30.8% of those who used Facebook once per day and fewer than 20% of infrequent users or non-users.

Caregiver-reported vaccination status (Table 1, Column 3) increased with caregiver education, ranging from 37.5% among caregivers with no formal schooling to 77.1% among those with an HND or higher education. Caregivers who recalled HPV vaccine messages were more likely to report that their adolescent had been vaccinated (82.4%**)** compared with caregivers who did not recall messages (55.0%**).**

### Multivariate analysis: Message recall and HPV vaccination

Adjusted odds ratios for caregiver recall of HPV vaccine messages are presented in Table 2, Column 1. After adjustment for socio-demographic characteristics, frequent Facebook use remained strongly associated with message recall. Caregivers who reported using Facebook multiple times per day had higher odds of recalling HPV vaccine messages (AOR = 7.59, 95% CI: 3.55–16.19), as did caregivers who reported daily Facebook use (AOR = 4.42, 95% CI: 1.39–14.04).

**Table 2:** Odds ratios from logistic regression associated with caregiver having seen information on the HPV vaccine and the adolescent getting vaccinated.

Variables	Caregiver saw information on the HPV vaccine (Column 1)	P value	Caregiver reported that adolescent was vaccinated (Column 2)	P value
**Age of caregiver**				
18-29	1.00		1.00	
30-39	0.88 (0.46–1.69)	0.699	1.40 (0.77–2.55)	0.264
40-49	0.73 (0.08–6.43)	0.778	1.28 (0.30–5.42)	0.734
**Gender of caregiver**				
Male	1.00		1.00	
Female	1.30 (0.22–7.48)	0.771	1.62 (0.40–6.57)	0.498
**Education of caregiver**				
No formal education	1.00		1.00	
Primary School Certificate	0.42 (0.24–0.73)	0.002	1.66 (0.68–4.04)	0.262
SSE/GCE	0.99 (0.66–1.49)	0.974	1.60 (0.32–6.82)	0.526
Ordinary National Diploma (OND)	0.74 (0.42–1.32)	0.306	4.15 (0.94–18.28)	0.060
Higher National Diploma (HND) or more	0.90 (0.33–2.45)	0.842	5.53 (1.45–21.08)	0.012
**Frequency of use of Facebook**				
Never	1.00		–	–
Infrequent	2.34 (0.30–18.43)	0.420	–	–
Once a day	4.42 (1.39–14.04)	0.012	–	–
Multiple times a day	7.59 (3.55–16.19)	<0.001	–	–
**Caregiver saw information on the HPV vaccine in the last 3 months**				
No	–		1.00	
Yes	–		3.36 (2.75–4.11)	<0.001
McFadden’s pseudo-R²	6.75%		11.56%	

Adjusted odds ratios for caregiver-reported adolescent HPV vaccination status are shown in Table 2, Column 2. Caregiver recall of HPV vaccine messages was associated with higher odds of reported vaccination (AOR = 3.36, 95% CI: 2.75–4.11). Model-predicted marginal effects derived from the logistic regression indicated a 23.3 percentage-point difference in the predicted probability of reported vaccination between caregivers who did and did not recall HPV vaccine messages (not shown). Caregiver education was also associated with reported vaccination status. Caregivers with an HND or higher education had higher odds of reporting that their adolescent was vaccinated compared with caregivers with no formal education (AOR = 5.53, 95% CI: 1.45–21.08). Confidence intervals for some education categories were wide, reflecting small sample sizes in certain strata. McFadden’s pseudo-R² values were 6.75% for the message recall model and 11.56% for the vaccination model, indicating modest but typical model fit for behavioral outcomes.

A two-stage residual inclusion (2SRI) analysis was conducted as a sensitivity check to assess potential endogeneity of message recall, using frequency of Facebook use as the instrument. The residual term from the first-stage model was not statistically significant (p = 0.736), suggesting no detectable endogeneity. However, this test does not establish instrument validity or rule out all sources of unobserved confounding.

### Service delivery statistics

Table 3. Shows estimated additional HPV vaccinations delivered during the July 16–20 MNCH week, by intervention status and area council.

**Table 3:** Estimated additional HPV vaccinations delivered during the July 16–20 MNCH week, by intervention status and area council.

Area council	Ward classification	Number of wards	Estimated population of girls aged 9–14	Number of HPV vaccinations delivered	Vaccinations per 1,000 girls
AMAC	Intervention wards	9	54,831	1,665*	30.4
AMAC	Control wards	3	54,831	—	—
Kuje	Intervention wards	6	6,857	222*	32.4
Kuje	Control wards	4	6,857	—	—

*Estimated additional vaccinations attributable to the digital campaign, based on ward-level regression analyses adjusting for ward population.

In ward-level regression analyses adjusting for ward population (not shown), intervention wards in AMAC delivered an estimated 185 more vaccinations per ward than control wards. When aggregated across the nine intervention wards, this difference corresponds to approximately 1,665 additional vaccinations associated with intervention wards, representing an estimated 3.0 percentage-point difference relative to the council-level denominator (54,831 girls aged 9–14).

In the Kuje Area Council, 984 HPV vaccinations were administered during MNCH week, corresponding to approximately 14.3% of the estimated population of girls aged 9–14 years (6,857). As in AMAC, this figure reflects overall service delivery during the recovery period rather than the effect of the digital intervention. In ward-level regression analyses adjusting for ward population (not shown), intervention wards in Kuje delivered an estimated 37 more vaccinations per ward than control wards. Aggregated across six intervention wards, this difference corresponds to approximately 222 additional vaccinations associated with intervention wards**,** representing an estimated 3.2 percentage-point difference relative to the council-level denominator (6,857 girls aged 9–14).The substantially higher vaccination proportion reported in the exit survey reflects selection of caregivers already engaged with health services and cumulative vaccination among facility attendees, rather than changes in population-level HPV vaccination delivery, which are better reflected in administrative coverage estimates.

## Discussion

### Summary of findings

This study found that caregiver recall of a digital market priming campaign was associated with higher caregiver-reported HPV vaccination among caregivers attending vaccination services during a brief resumption of service delivery following a prolonged disruption. The substantially higher vaccination proportion reported in the exit survey reflects selection of caregivers already engaged with health services and cumulative vaccination among facility attendees, rather than changes in population-level HPV vaccination delivery, which are better reflected in administrative coverage estimates.

### Comparison with prior evidence

These findings are comparable to prior evidence showing that health communication campaigns are often associated with modest but meaningful changes in health behaviors. Meta-analyses of mass media health communication interventions report behavioral differences of several percentage points across diverse outcomes, including vaccination, family planning, HIV prevention, and smoking cessation [9–11]. The magnitude of associations observed in this study is broadly consistent with this literature. In addition, recent quasi-experimental evidence from Bangladesh found that a behavioral insights–informed social media campaign promoting single-dose HPV vaccination was associated with a 5.3–9.5 percentage-point increase in vaccination rates, depending on the intervention approach used [19].

### Interpretation

Preparing caregivers to act during a brief resumption of services may help sustain programmatic momentum following service disruptions. In this study, digital priming messages were designed to address both motivational considerations and practical constraints by providing information about when and where HPV vaccination services were available during MNCH week. While motivation and ability were not directly measured, caregiver recall of messages and reported vaccination behavior suggest that digital communication may be associated with improved readiness to vaccinate when services become temporarily available. Conceptually, these findings suggest that digital communication strategies may help mitigate, though not eliminate, the disruptive effects of service interruptions.

The finding that the association between message recall and vaccination status was independent of caregiver education is noteworthy. Recall of social media messages occurred across multiple education levels, suggesting that digital campaigns may reach caregivers beyond those with the highest formal schooling. However, because the sample was drawn from caregivers already attending vaccination sites, these findings reflect reach among health-engaged populations rather than equitable population-wide access.

### Strengths and limitations

Because message exposure was measured through caregiver self-reported recall rather than verified exposure metrics, misclassification and recall bias are possible, and recall may reflect general attentiveness to health information rather than direct exposure to specific campaign content.

Although an instrumental variable analysis presented no evidence that unobserved factors correlated with Facebook use were driving the observed association between message recall and vaccination status, the exclusion restriction was imperfect. Frequency of Facebook use may be associated with vaccination behavior through pathways other than message recall, such as broader health information seeking or engagement with health services. Accordingly, the instrumental variable results should be interpreted as supportive sensitivity checks rather than definitive evidence of causal relationships. Because the intervention relied on digital advertising platforms, caregivers without regular internet or social media access were less likely to be exposed. This limitation underscores the importance of pairing digital priming strategies with complementary offline approaches to mitigate structural inequities in access.

### Implications for policy and research

These findings demonstrate the feasibility of using social media to support demand-shaping efforts during subnational service interruptions. Integrating behavioral insights–informed digital communication into broader immunization strategies may help programs maintain caregiver readiness during periods of limited service availability. Future research should examine how digital priming interacts with community-based mobilization, assess equity impacts, and evaluate the cost-effectiveness of subnational digital priming approaches to inform policy decisions.

## Conclusion

This study provides evidence that caregiver recall of a social media–based market priming strategy was associated with higher HPV vaccination activity during a brief resumption of services following a prolonged disruption. Recall of vaccine messages on Facebook or Instagram was associated with higher caregiver-reported adolescent vaccination among caregivers attending vaccination sites, and administrative data were consistent in direction, indicating modest differences in vaccination delivery between wards targeted by the digital campaign and comparison wards. While differences were modest, they were measurable during a highly constrained service window. If similar associations were observed at larger scale, even small percentage-point differences could translate into substantial numbers of additional girls receiving HPV vaccination. Overall, these findings suggest that behavioral insights–informed digital communication may represent a useful component of broader strategies to support HPV vaccination delivery during periods of service instability.

As LMICs scale up single-dose HPV vaccination programs, maintaining readiness despite interruptions in service delivery will be critical. Social media priming offers a low-cost, scalable tool to reach caregivers, enabling them to act quickly once the vaccine becomes available. Future studies should assess the cost-effectiveness of market priming strategies and examine how digital priming can be integrated with other delivery and communication strategies to maximize equity and impact.

## Supporting information

S1 Checklist Inclusivity in global research questionnaire. (DOCX)

S2 Checklist PLOS human subjects research checklist. (DOCX)
