# Supplementary material for: Leveraging social media to mitigate HPV vaccine service disruptions in Abuja, Nigeria
Source: PLoS One. 2026 Jul 9;21(7):e0352986. doi: 10.1371/journal.pone.0352986 (PMC13349177; doi:10.1371/journal.pone.0352986)
Supplement: Attachment — Submitted filename: 2025 12 28 Response to Reviewers.docx [file pone.0352986.s003.docx]

**Response to Reviewers**

**Manuscript ID:** PONE-D-25-50622
**Title:** Leveraging Social Media to Mitigate HPV Vaccine Service Disruptions in Abuja, Nigeria

Dear Dr. Muhammad,
Dear Reviewers,

We thank the Academic Editor and both reviewers for their thoughtful and constructive feedback. We are grateful for their suggestions to improve clarity, transparency, and interpretation. We have revised the manuscript substantially in response to these comments.

Below, we provide a **point-by-point response** to each comment. For ease of review, reviewer comments are reproduced in *italics*, followed by our responses. All changes have been incorporated into the revised manuscript and are highlighted in the tracked-changes version.

**REVIEWER #1**

We thank Reviewer #1 for their careful reading of the manuscript and for their constructive suggestions, which have helped us substantially improve clarity and transparency, particularly with respect to study design, sampling, and interpretation.

**Major Comment 1**

*“I wasn’t clear how the approach described would ‘ensure equitable reach beyond internet-connected populations’. Please expand on this…”*

**Response:**
We appreciate this comment and agree that the original wording could be interpreted as implying that the intervention itself ensured equitable reach. This was not our intention.

We have revised the Discussion and Conclusion to clarify that **digital priming strategies should be understood as complementary tools**, rather than standalone solutions, and that ensuring equitable reach requires integration with offline and community-based approaches. We now explicitly frame equity as a **design and implementation consideration**, not an achieved outcome of the present study. (“Insights-based subnational digital priming campaigns, when combined with complementary offline approaches that ensure equitable reach beyond internet-connected populations, may strengthen HPV vaccine introduction and program resilience.”)

**Major Comment 2**

*“I did not fully understand how the role of Facebook use was perceived… This needs clarification so that readers can judge the validity of your approach.”*

**Response:**
We appreciate this request for clarification and have revised the Statistical Analysis section to provide a clearer, non-technical explanation of the rationale for the instrumental variable (IV) approach and how it was interpreted.

Specifically, we now:

- Explain that the IV analysis was conducted as a **sensitivity check** to assess whether unobserved caregiver characteristics might bias the association between message recall and vaccination status.
- Clarify that frequency of Facebook use was used to predict recall in the first stage, and that residuals from this model were included in the outcome model to test for endogeneity.
- Explicitly acknowledge that frequency of Facebook use may be correlated with vaccination behavior through pathways other than recall, and therefore that the IV results should be interpreted **cautiously and as supportive rather than definitive**.

The revised paragraph in results is: “We tested for potential endogeneity of recall using a two-stage residual inclusion model with frequency of Facebook use as the instrument. The residual term from the first-stage model was not statistically significant (p = 0.736), indicating no evidence that unobserved factors correlated with Facebook use were driving the observed association between message recall and vaccination status.”

This is also explained more clearly in the limitations: “Although an instrumental variable analysis suggested no evidence that unobserved factors correlated with Facebook use were driving the observed association between message recall and vaccination status, the exclusion restriction was imperfect. Frequency of Facebook use may be associated with vaccination behavior through pathways other than message recall, such as broader health information seeking or engagement with health services. As such, the instrumental variable findings should be interpreted cautiously and as supportive rather than definitive.”

**Major Comment 3**

*“Please expand on how the numbers tie together and how the selection and recruitment of respondents was carried out…”*

**Response:**
We agree that greater transparency was needed and have revised the Methods and Results sections to clarify these points.

Specifically:

**(a)** We now clarify the meaning of campaign “reach,” explain why reported reach estimates differ across platforms and creatives, and specify total campaign exposure over the intervention period. We clarified the description of campaign reach and advertisement creatives to explain the difference between weekly reach estimates and reach per creative, defined ‘ad creatives,’ and noted that differences reflect overlap in exposure and platform-level reporting metrics rather than distinct audiences. (“During the campaign period, social media advertisements were delivered weekly to approximately 150,000 caregivers across targeted wards. A total of 30 distinct advertisement ‘creatives’—defined as unique combinations of images, text, and calls to action—were rotated during the campaign, each reaching an average of approximately 130,000 caregivers per week. Differences between these reach estimates reflect overlap in audience exposure across creatives, variation in delivery across Facebook and Instagram, and platform-level reporting metrics, rather than distinct or mutually exclusive audiences. As a result, cumulative reach over the campaign period exceeds weekly reach but does not represent unique individuals.”)

**(b)** We explicitly state that this study was **not a randomized controlled trial**. Intervention and control wards were defined based on geofenced delivery of social media advertisements, not random assignment. This clarification has been added to the Service Delivery Data sections (“Administrative data on the number of HPV vaccinations provided during the July 16–20 MNCH week were obtained from local government health authorities. In two area councils—the Abuja Municipal Area Council (AMAC, population 4.2 million) and Kuje Area Council (population 527,473)—wards were classified as intervention or control areas based on whether geofenced social media advertisements promoting HPV vaccination were delivered to caregivers residing in those wards. Assignment of wards to intervention or control status was based on programmatic considerations rather than randomization.”)

**(c)** We clarify that not all caregivers in intervention wards necessarily received or recalled messages, and that ward-level analyses assume partial exposure (“Not all caregivers residing in intervention wards were necessarily exposed to or recalled the digital messages. Ward-level comparisons therefore reflect differences in vaccination uptake in areas targeted by the digital campaign, rather than individual-level exposure effects.”)

**(d–e)** We provide additional detail on how eligibility was determined, how systematic sampling was implemented, and how field supervisors monitored adherence (“Within each data collection site, interviewers used systematic sampling, approaching every second eligible caregiver. Eligibility was determined by confirming that the respondent was a caregiver of a girl aged 9–17 years who had attended the facility or pharmacy during the MNCH week. Field supervisors monitored interviewer adherence to sampling procedures through spot checks and real-time review of submitted survey forms.”)

**(f–g)** In study limitations, we acknowledge potential selection and response biases associated with recruitment at pharmacies and the absence of response-rate data, and we discuss how these may affect interpretation (“Finally, because participants were recruited at health facilities and pharmacies, the sample may over-represent caregivers who are more health-engaged, potentially limiting generalizability.”).

**(h)** We have added a new table, Table 3, summarizing service delivery statistics for intervention and control wards to improve transparency and provided a clearer explanation of the findings. (“Table 3. Shows estimated additional HPV vaccinations delivered during the July 16–20 MNCH week, by intervention status and area council. Administrative data showed that 6,516 HPV vaccinations were administered in the Abuja Municipal Area Council (AMAC) during the July 16–20 MNCH week, corresponding to vaccination of approximately 11.9% of the estimated population of girls aged 9–14 (54,831). This figure reflects overall service delivery during the recovery period rather than the effect of the digital intervention. In ward-level regression analyses adjusting for ward population (not shown), intervention wards in AMAC delivered an estimated 185 more vaccinations per ward than control wards. Multiplying this per-ward difference by the nine intervention wards yields an estimated 1,665 additional vaccinations attributable to the campaign, corresponding to an approximately 3.0-percentage-point increase relative to the council-level denominator (54,831 girls aged 9–14). In the Kuje area council, administrative data indicated that 984 HPV vaccinations were administered during the July 16–20 MNCH week, corresponding to vaccination of approximately 14.3% of the estimated population of girls aged 9–14 (6,857). As in AMAC, this figure reflects overall service delivery during the recovery period rather than the effect of the digital intervention. In ward-level regression analyses adjusting for ward population (not shown), intervention wards delivered an estimated 37 more vaccinations per ward than control wards. Multiplying this per-ward difference by the six intervention wards yields an estimated 222 additional vaccinations attributable to the campaign, corresponding to an approximately 3.2-percentage-point increase relative to the council-level denominator (6,857 girls aged 9–14).”)

**Major Comment 4**

*“More limitations should be included…”*

**Response:**
We thank the reviewer for these suggestions and have expanded the Limitations section to explicitly address:

- Potential selection bias related to pharmacy-based recruitment and systematic sampling
- Possible response bias
- Potential spillover or cross-fertilization between intervention and control wards
- The likely direction of recall and social desirability bias, which may overestimate the strength of associations

(“ The study’s strengths include the use of both caregiver exit survey data and administrative service delivery statistics, which provided convergent evidence for the observed associations. However, several limitations should be noted. Data on both adolescent vaccination status and exposure to the social media campaign were caregiver-reported, introducing the potential for response bias, including recall and social desirability biases, which may have led to overestimation of the strength of observed associations. In addition, the cross-sectional study design precludes causal inference.

Although an instrumental variable analysis presented no evidence that unobserved factors correlated with Facebook use were driving the observed association between message recall and vaccination status, the exclusion restriction was imperfect. Frequency of Facebook use may be associated with vaccination behavior through pathways other than message recall, such as broader health information seeking or engagement with health services. As such, the instrumental variable findings should be interpreted cautiously and as supportive rather than definitive.

Finally, because participants were recruited at health facilities and pharmacies using systematic sampling, the sample may over-represent caregivers who are more health-engaged, potentially limiting generalizability. In addition, spillover or cross-fertilization of messages between intervention and control wards—through interpersonal communication or exposure to shared media environments—may have attenuated observed differences between study groups.”

**Minor Comments**

1. **Scope beyond service disruption:**
   We clarify that the intervention was designed for a period of service disruption, while noting its potential relevance for broader HPV vaccination efforts (“Although the intervention was designed to support HPV vaccine uptake during a temporary service disruption, the findings may also inform demand-generation strategies for routine HPV vaccination when services are available more consistently.”).
2. **Mechanism of action:**
   We now explicitly state that messages helped caregivers know when and where vaccination services were available, in addition to increasing motivation (“In addition to increasing motivation, the messages provided practical information about when and where HPV vaccination services were available during MNCH week, helping caregivers translate intent into action.”).
3. **Message content:**
   We expanded the manuscript to include a clearer description of the content and engagement strategies used in the social media advertisements. Specifically, we describe how messages addressed common caregiver concerns, featured trusted messengers such as pharmacists, emphasized the limited availability of services during MNCH week, and encouraged caregivers to sign up to receive notifications when vaccination services became available. This description is now included in the Introduction as part of the campaign description. (“**Message content and engagement strategy.** The digital market priming campaign employed multiple short-form social media advertisements informed by prior behavioral insights research and iterative testing. Advertisements featured locally relevant themes and trusted messengers, including community pharmacists, and were designed to address common caregiver concerns about HPV vaccination, such as safety, cost, eligibility, and fertility-related misconceptions. In response to service disruptions and uncertainty about vaccine availability, a subset of advertisements encouraged caregivers to sign up to receive notifications when HPV vaccination services became available in their area. This approach allowed the campaign to maintain engagement during periods of service interruption and enabled follow-up messaging when vaccination opportunities arose. Messages consistently emphasized that HPV vaccination was free, time-limited, and available for girls aged 9–14 years, and provided information on where services could be accessed during MNCH week. Multiple creatives were rotated and adapted over time to improve relevance and engagement while maintaining consistent core themes related to motivation, trust, and caregivers’ ability to act. Caregiver recall of these messages was later assessed in the exit survey and used as the primary exposure variable in individual-level analyses.")
4. **Instagram use:**
   We clarify that Facebook and Instagram ads were delivered jointly through Meta’s advertising platform and that platform-specific exposure was not measured separately (“Advertisements were delivered jointly across Facebook and Instagram using Meta’s advertising platform; platform-specific exposure could not be distinguished and was therefore not measured separately.”)
5. **Caregiver-reported vaccination status:**
   We have made the suggested change in the Conclusion. (“A caregiver’s recall of vaccine messages on Facebook was strongly associated with caregiver-reported vaccination status.”)

**REVIEWER #2**

We thank Reviewer #2 for their positive assessment and helpful suggestions.

**Vaccination status:** We now more explicitly acknowledge the limitations of caregiver-reported vaccination status and clarify that no verification via vaccination cards or facility records was attempted (“Data on both adolescent vaccination status and exposure to the social media campaign were caregiver-reported, introducing the potential for response bias, including recall and social desirability biases, which may have led to overestimation of the strength of observed associations. In addition, the cross-sectional study design precludes causal inference.”)

- **Digital reach and representativeness:** That the intervention relies on Facebook and Instagram may exclude caregivers without access to these platforms has been addressed. (“As the intervention relied on digital advertising platforms, caregivers without regular internet or social media access may have been less likely to be exposed, underscoring the need to pair digital priming strategies with complementary offline approaches to promote equitable reach.”}
- **Recruitment through pharmacies:** We clarified the process for selecting private pharmacies used for caregiver recruitment by specifying that pharmacies were drawn from the WellaHealth partner network and had received targeted training on HPV vaccination counseling. (“Caregivers recruited through private pharmacies were approached at the pharmacy following service use. These pharmacies were purposively selected based on their participation in a WellaHealth-supported pharmacist training initiative focused on HPV vaccination counseling.”) We also added this as a limitation (“Because pharmacy-based recruitment was limited to WellaHealth partner pharmacies trained in HPV counseling, caregivers recruited through this pathway may not be representative of all pharmacy clients.”)
- **Table 1 discrepancy:** We have corrected the minor inconsistency in Table 1 totals. The number of respondents ages 40 and older were 88 (instead of 89).

**Editorial and Journal Requirements**

We have addressed all additional editorial requirements, including:

- Expanded ethics statement specifying informed consent procedures, type of consent, and parental consent
- Submission of the completed PLOS ONE inclusivity in global research questionnaire as Supporting Information
- Correction of inconsistencies in funding information
- Formatting updates to meet PLOS ONE style requirements

We sincerely thank the editor and reviewers for their constructive feedback, which has significantly strengthened the manuscript. We hope that the revised version meets the journal’s publication criteria and we look forward to your further consideration.

Sincerely,
**Sohail Agha, PhD**
(on behalf of all authors)
