# Supplementary material for: Leveraging social media to mitigate HPV vaccine service disruptions in Abuja, Nigeria
Source: PLoS One. 2026 Jul 9;21(7):e0352986. doi: 10.1371/journal.pone.0352986 (PMC13349177; doi:10.1371/journal.pone.0352986)
Supplement: Attachment — Submitted filename: 2026 1 18 Response to Reviewers.docx [file pone.0352986.s004.docx]

**Response to Reviewers**

Manuscript ID: **PONE-D-25-50622R1**
Title: *Leveraging Social Media to Mitigate HPV Vaccine Service Disruptions in Abuja, Nigeria*

Dear Dr. Muhammad and Reviewers,

We thank the Academic Editor and reviewers for their careful review and constructive feedback. We appreciate the positive assessment of the study’s rigor and the clear guidance provided. We have revised the manuscript extensively to address all comments and to improve clarity, transparency, and alignment with the observational nature of the study. Below, we provide a detailed, point-by-point response indicating how each comment was addressed. Page and section references refer to the revised manuscript.

**Abstract**

**Reviewer comment:**
The period of the health worker strike lacks a specific year.

**Response:**
We have specified the year throughout the Abstract and manuscript. The Abstract now states that the strike occurred in 2025, with services restored during July 16–20, 2025 MNCH week .

**Reviewer comment:**
The phrase “post-test-only exit survey” may imply an experimental design.

**Response:**
We removed this phrasing and clarified that the study used a cross-sectional exit survey with an observational design. No causal language is used in the Abstract or elsewhere.

**Reviewer comment:**
Clarify the purpose of the two-stage residual inclusion (2SRI) model.

**Response:**
The Abstract now explicitly states that the 2SRI model was used as a sensitivity analysis to assess potential bias from unobserved confounding, not as a causal estimator.

**Reviewer comment:**
The reported percentage-point differences lack explanation.

**Response:**
We clarified that the 23.3 percentage-point estimate reflects model-predicted marginal effects, and that the 7.9 percentage-point difference represents an application of those marginal effects to the study population .

**Reviewer comment:**
“Administrative data corroborated” may imply causal validation.

**Response:**
We replaced this wording with “administrative data were consistent with survey findings” throughout the manuscript,

**Reviewer comment:**
The concluding claim overstates program-level impact.

**Response:**
We softened the conclusion to reflect association rather than impact, stating that digital priming *may help maintain caregiver readiness* rather than strengthen programs directly

**Introduction**

**Reviewer comment:**
The Introduction lacks a clear study objective.

**Response:**
We added a clear objective sentence at the end of the Introduction stating that the study assessed whether caregiver recall of a digital market priming campaign was associated with HPV vaccine uptake during a brief service resumption in Abuja, Nigeria.

**Reviewer comment:**
The discussion of “5–10 percentage point behavior change” could be misinterpreted as a benchmark.

**Response:**
We clarified that these figures are contextual estimates from prior studies, not expected or benchmark effects for this study.

**Reviewer comment:**
Campaign delivery metrics belong in Methods rather than Introduction.

**Response:**
All implementation details (reach, creatives, delivery) were moved to a dedicated “Message Content and Engagement Strategy” subsection in Methods. The Introduction now focuses on conceptual rationale only.

**Reviewer comment:**
Define “market priming strategies” operationally.

**Response:**
We added a concise operational definition tailored to HPV vaccination and service disruptions.

**Reviewer comment:**
Clarify how the Fogg Behavior Model relates to measured variables.

**Response:**
We now explicitly state that the model informed message design, while message recall and vaccination status are observable indicators, not direct measures of motivation or ability.

**Reviewer comment:**
Specify timing of strike, campaign, and MNCH week.

**Response:**
Exact months and dates are now provided consistently throughout the Introduction and Methods .

**Methods**

**Reviewer comment:**
Clarify whether survey and administrative data were linked.

**Response:**
We clarified that data linkage was **ecological and ward-level**, with no individual identifiers matched across data sources .

**Reviewer comment:**
Provide sample size justification.

**Response:**
We added a sample size statement indicating that 300 interviews were targeted, providing ±6 percentage-point precision accounting for clustering (design effect = 1.5), but that 271 interviews were achieved due to the short service window.

**Reviewer comment:**
Address heterogeneity introduced by private pharmacies.

**Response:**
We clarified the role of pharmacies, described their selection, and noted that analyses accounted for clustering by recruitment location.

**Reviewer comment:**
Acknowledge recall bias due to narrow data collection window.

**Response:**
Recall bias is now acknowledged explicitly in the Discussion, noting that proximity to campaign activity may inflate recall.

**Reviewer comment:**
Verify ethical approval dates.

**Response:**
Ethical approval information was reviewed and aligned. The protocol number and approval date now match the NHREC documentation.

**Reviewer comment:**
Facebook use frequency may violate the exclusion restriction.

**Response:**
We explicitly acknowledge this limitation, stating that the instrument may be correlated with health-seeking behavior, and that the 2SRI analysis should be interpreted as a sensitivity check only.

**Reviewer comment:**
Specify why 2SRI was used instead of 2SLS.

**Response:**
We clarified that 2SRI was used because the outcome variable is binary, making it appropriate for nonlinear models.

**Reviewer comment:**
Clarify non-random assignment of intervention wards.

**Response:**
We explicitly state that ward assignment was non-random and programmatic, and that ward-level analyses are quasi-experimental and observational.

**Reviewer comment:**
Acknowledge uncertainty in population denominators.

**Response:**
We added a statement noting uncertainty associated with population projections and UN-derived age proportions .

**Reviewer comment:**
Address missing data and model diagnostics.

**Response:**
We added a paragraph stating that no missing data were observed, that multicollinearity was assessed using variance inflation factors, and that standard goodness-of-fit statistics were used. Significance thresholds (α = 0.05, two-tailed tests) are now specified.

**Results**

**Reviewer comment:**
Table 1 column headings are unclear.

**Response:**
Table 1 headings were revised to clearly distinguish p-values for message recall and vaccination status.

**Reviewer comment:**
High recall rate may reflect misclassification or recall bias.

**Response:**
We explicitly acknowledge the possibility of recall bias and exposure misclassification arising from self-reported message recall in the Results and Discussion. We note that recall may reflect broader health engagement rather than verified exposure and interpret findings accordingly.

**Reviewer comment:**
Wide confidence intervals should be noted.

**Response:**
We added a sentence noting that wide confidence intervals reflect sparse data in certain strata.

**Reviewer comment:**
Clarify marginal effects interpretation.

**Response:**
We explicitly state that percentage-point differences are model-predicted marginal effects, not direct differences in observed proportions.

**Reviewer comment:**
“Attributable to the campaign” is causal in tone.

**Response:**
We replaced this phrasing with “associated with intervention wards” throughout the Results and Discussion.

**Reviewer comment:**
Explain discrepancy between survey and administrative coverage.

**Response:**
We added explicit clarification that survey vaccination reflects facility-attending caregivers and cumulative vaccination, whereas administrative data reflect population-level delivery.

**Discussion and Conclusion**

**Reviewer comment:**
Avoid causal language throughout.

**Response:**
We carefully reviewed and revised the Discussion and Conclusion to consistently use associational language, avoiding claims of causality or program-level impact.

**Reviewer comment:**
Clarify that administrative data agreement is directional, not magnitude-based.

**Response:**
We now state that administrative data were consistent in direction, not magnitude.

**Reviewer comment:**
Add qualifier to national-scale implications.

**Response:**
We added qualifiers such as “if replicated at larger scale” when discussing potential population impact .

**Reviewer comment:**
Strengthen equity discussion.

**Response:**
We explicitly note that the sample over-represents health-engaged caregivers and that digital strategies should be paired with offline approaches to mitigate access inequities.

We believe these revisions have strengthened the manuscript and aligned it fully with PLOS ONE’s standards for transparency and observational research. We thank the Editor and reviewers again for their valuable guidance and hope the revised manuscript is now suitable for publication.

Sincerely,
**Sohail Agha, PhD**
(on behalf of all authors)
