# Supplementary material for: Leveraging social media to mitigate HPV vaccine service disruptions in Abuja, Nigeria
Source: PLoS One. 2026 Jul 9;21(7):e0352986. doi: 10.1371/journal.pone.0352986 (PMC13349177; doi:10.1371/journal.pone.0352986)
Supplement: Attachment — Submitted filename: 2026 6 12 Response_to_Reviewers_R3.docx [file pone.0352986.s005.docx]

**Response to Reviewers**

**Manuscript PONE-D-25-50622R2**

*Leveraging social media to mitigate HPV vaccine service disruptions in Abuja, Nigeria*

Dear Dr. Muhammad,

Thank you for your positive assessment of our revised manuscript and for the opportunity to address the remaining points. We are grateful for your careful reading. Below we respond to each item in turn. Changes to the manuscript are shown using tracked changes in the revised file.

**1. NHREC protocol number consistency**

**Editor comment:**

*The NHREC protocol number does not match across the submission. The Ethics Statement gives NHREC/01/01/2007-15/07/2024, approved 15 July 2024, while the Methods section gives NHREC/01/01/2007-17/08/2024 with no date. Please check both against the approval letter and make the form and the manuscript agree.*

**Response:**

We thank the editor for catching this discrepancy. We checked both entries against the NHREC approval letter. The letter specifies two distinct identifiers: a Protocol Number (NHREC/01/01/2007-17/08/2024, corresponding to the date of receipt of the valid application) and an Approval Number (NHREC/01/01/2007-29/08/2024, corresponding to the date of final determination). The approval was granted on 29 August 2024 and is valid through 28 August 2025. The number previously stored in the submission system Ethics Statement field (NHREC/01/01/2007-15/07/2024, 15 July 2024) was a transcription error and does not appear in the approval letter.

To resolve the inconsistency, we have revised the Ethical Considerations section of the manuscript to state both identifiers and the approval and validity dates explicitly. The Methods section now reads:

*The study protocol, including the consent process, was reviewed and approved by the National Health Research Ethics Committee of Nigeria (NHREC) (protocol number NHREC/01/01/2007-17/08/2024; approval number NHREC/01/01/2007-29/08/2024), approved on 29 August 2024 and valid through 28 August 2025 [16].*

We have also corrected the Ethics Statement field in the submission system to match this text, so that the form and the manuscript now agree and both correspond to the approval letter.

**2. Abstract stored in the submission system**

**Editor comment:**

*The abstract stored in the submission system is still the earlier version. For example, it reads 33.8%, “assesses the effect,” “to test endogeneity,” and “corroborated,” while the abstract in the manuscript file is the revised one. Production uses the manuscript abstract, but you may want to update the system field so the two match.*

**Response:**

Thank you. We have replaced the abstract stored in the submission system with the current revised abstract from the manuscript file, so that the two versions now match. The system field no longer contains the earlier wording (e.g., “33.8%,” “assesses the effect,” “to test endogeneity,” or “corroborated”). No change to the manuscript was required for this item, as the manuscript already contained the revised abstract.

**3. Confidence interval in Table 2 (recall and vaccination)**

**Editor comment:**

*The confidence interval for the recall and vaccination association in Table 2 (AOR 3.36, 95% CI 2.75 to 4.11) is narrower than I would expect given the sample size and the other estimates in the model. Please confirm the model and the interval. If it is correct as reported, leave it as is.*

**Response:**

We re-estimated the model and confirm the coefficient and interval are correct as reported. The relatively narrow interval reflects the strong and precisely estimated association between recall and vaccination (z = 11.82), in contrast to the wider intervals on the sparser sociodemographic categories. We have left the estimate unchanged.

We believe these revisions fully address the remaining points. Thank you again for your time and for the constructive review of our manuscript.

Sincerely,

Sohail Agha, on behalf of all co-authors
