# Supplementary material for: Leveraging social media to mitigate HPV vaccine service disruptions in Abuja, Nigeria
Source: PLoS One. 2026 Jul 9;21(7):e0352986. doi: 10.1371/journal.pone.0352986 (PMC13349177; doi:10.1371/journal.pone.0352986)
Supplement: Peer review history

# Peer review: Decision Letter 0

**DOI:** 10.1371/journal.pone.0352986.r001

**Authors:** Ameer Muhammad

22 Dec 2025

PONE-D-25-50622

Leveraging Social Media to Mitigate HPV Vaccine Service Disruptions in Abuja, Nigeria

PLOS One

Dear Dr. Agha,

Thank you for submitting your manuscript to PLOS ONE. After careful consideration, we feel that it has merit but does not fully meet PLOS ONE’s publication criteria as it currently stands. Therefore, we invite you to submit a revised version of the manuscript that addresses the points raised during the review process.

Please submit your revised manuscript by Feb 05 2026 11:59PM. If you will need more time than this to complete your revisions, please reply to this message or contact the journal office at plosone@plos.org. When you're ready to submit your revision, log on to https://www.editorialmanager.com/pone/ and select the 'Submissions Needing Revision' folder to locate your manuscript file.

Please include the following items when submitting your revised manuscript:

- A letter that responds to each point raised by the academic editor and reviewer(s). You should upload this letter as a separate file labeled 'Response to Reviewers'.
- A marked-up copy of your manuscript that highlights changes made to the original version. You should upload this as a separate file labeled 'Revised Manuscript with Track Changes'.
- An unmarked version of your revised paper without tracked changes. You should upload this as a separate file labeled 'Manuscript'.

If you would like to make changes to your financial disclosure, please include your updated statement in your cover letter. Guidelines for resubmitting your figure files are available below the reviewer comments at the end of this letter.

If applicable, we recommend that you deposit your laboratory protocols in protocols.io to enhance the reproducibility of your results. Protocols.io assigns your protocol its own identifier (DOI) so that it can be cited independently in the future. For instructions see: https://journals.plos.org/plosone/s/submission-guidelines#loc-laboratory-protocols. Additionally, PLOS ONE offers an option for publishing peer-reviewed Lab Protocol articles, which describe protocols hosted on protocols.io. Read more information on sharing protocols at https://plos.org/protocols?utm_medium=editorial-email&utm_source=authorletters&utm_campaign=protocols.

We look forward to receiving your revised manuscript.

Kind regards,

Ameer Muhammad

Academic Editor

PLOS One

Journal Requirements:

When submitting your revision, we need you to address these additional requirements.

1.Please ensure that your manuscript meets PLOS ONE's style requirements, including those for file naming. The PLOS ONE style templates can be found at

https://journals.plos.org/plosone/s/file?id=wjVg/PLOSOne_formatting_sample_main_body.pdf and

https://journals.plos.org/plosone/s/file?id=ba62/PLOSOne_formatting_sample_title_authors_affiliations.pdf

2. Please provide additional details regarding participant consent. In the ethics statement in the Methods and online submission information, please ensure that you have specified (1) whether consent was informed and (2) what type you obtained (for instance, written or verbal, and if verbal, how it was documented and witnessed). If your study included minors, state whether you obtained consent from parents or guardians. If the need for consent was waived by the ethics committee, please include this information.

3. Please include a complete copy of PLOS’ questionnaire on inclusivity in global research in your revised manuscript. Our policy for research in this area aims to improve transparency in the reporting of research performed outside of researchers’ own country or community. The policy applies to researchers who have travelled to a different country to conduct research, research with Indigenous populations or their lands, and research on cultural artefacts. The questionnaire can also be requested at the journal’s discretion for any other submissions, even if these conditions are not met. Please find more information on the policy and a link to download a blank copy of the questionnaire here: https://journals.plos.org/plosone/s/best-practices-in-research-reporting. Please upload a completed version of your questionnaire as Supporting Information when you resubmit your manuscript.

4. We note that the grant information you provided in the ‘Funding Information’ and ‘Financial Disclosure’ sections do not match.

When you resubmit, please ensure that you provide the correct grant numbers for the awards you received for your study in the ‘Funding Information’ section.

5. If the reviewer comments include a recommendation to cite specific previously published works, please review and evaluate these publications to determine whether they are relevant and should be cited. There is no requirement to cite these works unless the editor has indicated otherwise.

[Note: HTML markup is below. Please do not edit.]

Reviewers' comments:

Reviewer's Responses to Questions

**Comments to the Author**

1. Is the manuscript technically sound, and do the data support the conclusions?

The manuscript must describe a technically sound piece of scientific research with data that supports the conclusions. Experiments must have been conducted rigorously, with appropriate controls, replication, and sample sizes. The conclusions must be drawn appropriately based on the data presented.

Reviewer #1: Partly

Reviewer #2: Yes

**********

2. Has the statistical analysis been performed appropriately and rigorously?

Reviewer #1: I Don't Know

Reviewer #2: Yes

**********

3. Have the authors made all data underlying the findings in their manuscript fully available?

The PLOS Data policy requires authors to make all data underlying the findings described in their manuscript fully available without restriction, with rare exception (please refer to the Data Availability Statement in the manuscript PDF file). The data should be provided as part of the manuscript or its supporting information, or deposited to a public repository. For example, in addition to summary statistics, the data points behind means, medians and variance measures should be available. If there are restrictions on publicly sharing data—e.g. participant privacy or use of data from a third party—those must be specified.

Reviewer #1: No

Reviewer #2: Yes

**********

4. Is the manuscript presented in an intelligible fashion and written in standard English?

PLOS ONE does not copyedit accepted manuscripts, so the language in submitted articles must be clear, correct, and unambiguous. Any typographical or grammatical errors should be corrected at revision, so please note any specific errors here.

Reviewer #1: Yes

Reviewer #2: Yes

**********

5. Review Comments to the Author

Please use the space provided to explain your answers to the questions above. You may also include additional comments for the author, including concerns about dual publication, research ethics, or publication ethics. (Please upload your review as an attachment if it exceeds 20,000 characters)

Reviewer #1: There are gaps and some possible inconsistencies in the write-up of methods so did not feel could be sure about the rigour

The motivation for the exercise is clear and if social media can boost vaccination rates substantially it will be a useful tool so I applaud the attempt to measure this.

My comments largely seek clarity as I could not piece all the story together

Major:

1. I wasn’t clear how the approach described would “ensure equitable reach beyond internet-connected populations”. Please expand on this as it changes the reader’s idea of potential impact of social media.

2. I did not fully understand how the role of Facebook use was perceived. I understand that you included it as an instrumental variable and the reason for having an instrumental variable. I am not a statistician and so would appreciate a fuller explanation. Is it that the residuals from the first stage are not correlated with the outcome? You state under Statistical Analysis that you assumed that frequency of Facebook use would be strongly correlated with recall but not with vaccine uptake but it is correlated with the latter. This needs clarification so that readers can judge the validity of your approach.

3. Please expand on how the numbers tie together and how the selection and recruitment of respondents was carried out. Transparency is important for credibility and understanding of the results and for the scope for drawing conclusions.

a. In the last para of the Introduction you say 150000 caregivers were reached each week and 30 different ad creatives (what is a creative?) reached an average of 130000 caregivers per week. So how many in total over the whole campaign and why is the second number different from the first?

b. Then you refer to 3 councils only (a subset of the area in which the messages were sent out?) and that participants were selected randomly but later under Service Delivery Data say that there were intervention and control areas. Thus it seems there was a randomised control trial. If so, please spell this out under the paragraph on sampling ..was the selection stratified by intervention or control? If not a trial please explain how the paragraph on Service Delivery Data fits into the research design.

c. You compare numbers vaccinated in intervention vs control areas in 2 admin areas. Would everyone in the intervention area have been sent the messages or are you thinking that the word would have spread out from a sample who were sent the messages to the rest of caregivers in the intervention areas?

d. How was the random sampling supervised? How was it determined which individuals were eligible (i.e. i. a caregiver and ii. the second eligible caregiver)?

e. Did selection take place before people visited pharmacies or at the pharmacies?

f. The fact that all those participating visited pharmacies suggests that they had a level of health consciousness and there could be a selection bias as those who did not visit pharmacies may also have been less likely to take any notice of the messages.

g. There is no information on response rates and any data you have on characteristic of those who did and did not take part.. There could be selection bias and response bias distorting your information. Can you give a flow chart showing how you got from the number sent messages to the number interviewed?

h. Generally the Service Delivery statistics need more explanation and a table of the results would probably make them more transparent than trying to limit a textual account of the results.

4. More limitations should be included:

a. Possible selection bias: from being in pharmacies; from the method of selecting every 2nd eligible caregiver

b. Possible response bias

c. Cross-fertilisation between those sent the messages and those who were not (could this have taken place?)

d. Say more about possible effect of recall bias and social desirability bias on estimates (I think would tend to over-estimation of strength of association)

Minor:

1. The period researched covered a brief resumẻ of vaccination after a gap of several months. I believe that the intervention was designed specifically for this situation but were you also seeing this as a step towards a more general use in mind aiming to increase HPV vaccination rates?

2. I was not clear why receiving a message made it easier for caregivers to access vaccination.. was this just that they would know when and where to go to get it done?

3. Could you give the message in an appendix? This would help make the study replicable and help the reader think about how it might be motivating.

4. Why wasn’t Instagram use measured?

5. In the Conclusion line 4 please spell out that it is the caregiver’s report of vaccination status. I know the next sentence clarifies this but I suggest being as explicit as possible throughout.

Reviewer #2: I would like to thank the authors for a clear and timely manuscript addressing an important implementation challenge for HPV vaccination. I have a few brief comments:

Vaccination status: Vaccination status is caregiver-reported rather than verified using vaccination cards or facility records, which may introduce recall or social desirability bias. It would be helpful to acknowledge this more explicitly and clarify whether any verification was attempted.

Digital reach and representativeness: The intervention relies on Facebook and Instagram, which may exclude caregivers without access to these platforms. A short discussion on how this may affect equity and generalizability would strengthen the interpretation of the findings.

Recruitment through private pharmacies: Caregivers were also recruited via private pharmacies, but the process for selecting these pharmacies is not fully described. Additional clarification on this recruitment pathway would improve transparency.

Minor inconsistency in Table 1: In Table 1, the totals for caregiver age appear to sum to 272, while the reported sample size is 271. The authors may wish to review and correct this minor discrepancy.

**********

6. PLOS authors have the option to publish the peer review history of their article (what does this mean?). If published, this will include your full peer review and any attached files.

If you choose “no”, your identity will remain anonymous but your review may still be made public.

**Do you want your identity to be public for this peer review?**  For information about this choice, including consent withdrawal, please see our Privacy Policy.

Reviewer #1: **Yes:**  Elizabeth Breeze

Reviewer #2: No

**********

[NOTE: If reviewer comments were submitted as an attachment file, they will be attached to this email and accessible via the submission site. Please log into your account, locate the manuscript record, and check for the action link "View Attachments". If this link does not appear, there are no attachment files.]

To ensure your figures meet our technical requirements, please review our figure guidelines: https://journals.plos.org/plosone/s/figures

You may also use PLOS’s free figure tool, NAAS, to help you prepare publication quality figures: https://journals.plos.org/plosone/s/figures#loc-tools-for-figure-preparation.

NAAS will assess whether your figures meet our technical requirements by comparing each figure against our figure specifications.

# Author comment: Author response to Decision Letter 1

**DOI:** 10.1371/journal.pone.0352986.r002

30 Dec 2025

Response to Reviewers

Manuscript ID: PONE-D-25-50622

Title: Leveraging Social Media to Mitigate HPV Vaccine Service Disruptions in Abuja, Nigeria

Dear Dr. Muhammad,

Dear Reviewers,

We thank the Academic Editor and both reviewers for their thoughtful and constructive feedback. We are grateful for their suggestions to improve clarity, transparency, and interpretation. We have revised the manuscript substantially in response to these comments.

Below, we provide a point-by-point response to each comment. For ease of review, reviewer comments are reproduced in italics, followed by our responses. All changes have been incorporated into the revised manuscript and are highlighted in the tracked-changes version.

REVIEWER #1

We thank Reviewer #1 for their careful reading of the manuscript and for their constructive suggestions, which have helped us substantially improve clarity and transparency, particularly with respect to study design, sampling, and interpretation.

Major Comment 1

“I wasn’t clear how the approach described would ‘ensure equitable reach beyond internet-connected populations’. Please expand on this…”

Response:

We appreciate this comment and agree that the original wording could be interpreted as implying that the intervention itself ensured equitable reach. This was not our intention.

We have revised the Discussion and Conclusion to clarify that digital priming strategies should be understood as complementary tools, rather than standalone solutions, and that ensuring equitable reach requires integration with offline and community-based approaches. We now explicitly frame equity as a design and implementation consideration, not an achieved outcome of the present study. (“Insights-based subnational digital priming campaigns, when combined with complementary offline approaches that ensure equitable reach beyond internet-connected populations, may strengthen HPV vaccine introduction and program resilience.”)

Major Comment 2

“I did not fully understand how the role of Facebook use was perceived… This needs clarification so that readers can judge the validity of your approach.”

Response:

We appreciate this request for clarification and have revised the Statistical Analysis section to provide a clearer, non-technical explanation of the rationale for the instrumental variable (IV) approach and how it was interpreted.

Specifically, we now:

• Explain that the IV analysis was conducted as a sensitivity check to assess whether unobserved caregiver characteristics might bias the association between message recall and vaccination status.

• Clarify that frequency of Facebook use was used to predict recall in the first stage, and that residuals from this model were included in the outcome model to test for endogeneity.

• Explicitly acknowledge that frequency of Facebook use may be correlated with vaccination behavior through pathways other than recall, and therefore that the IV results should be interpreted cautiously and as supportive rather than definitive.

The revised paragraph in results is: “We tested for potential endogeneity of recall using a two-stage residual inclusion model with frequency of Facebook use as the instrument. The residual term from the first-stage model was not statistically significant (p = 0.736), indicating no evidence that unobserved factors correlated with Facebook use were driving the observed association between message recall and vaccination status.”

This is also explained more clearly in the limitations: “Although an instrumental variable analysis suggested no evidence that unobserved factors correlated with Facebook use were driving the observed association between message recall and vaccination status, the exclusion restriction was imperfect. Frequency of Facebook use may be associated with vaccination behavior through pathways other than message recall, such as broader health information seeking or engagement with health services. As such, the instrumental variable findings should be interpreted cautiously and as supportive rather than definitive.”

Major Comment 3

“Please expand on how the numbers tie together and how the selection and recruitment of respondents was carried out…”

Response:

We agree that greater transparency was needed and have revised the Methods and Results sections to clarify these points.

Specifically:

(a) We now clarify the meaning of campaign “reach,” explain why reported reach estimates differ across platforms and creatives, and specify total campaign exposure over the intervention period. We clarified the description of campaign reach and advertisement creatives to explain the difference between weekly reach estimates and reach per creative, defined ‘ad creatives,’ and noted that differences reflect overlap in exposure and platform-level reporting metrics rather than distinct audiences. (“During the campaign period, social media advertisements were delivered weekly to approximately 150,000 caregivers across targeted wards. A total of 30 distinct advertisement ‘creatives’—defined as unique combinations of images, text, and calls to action—were rotated during the campaign, each reaching an average of approximately 130,000 caregivers per week. Differences between these reach estimates reflect overlap in audience exposure across creatives, variation in delivery across Facebook and Instagram, and platform-level reporting metrics, rather than distinct or mutually exclusive audiences. As a result, cumulative reach over the campaign period exceeds weekly reach but does not represent unique individuals.”)

(b) We explicitly state that this study was not a randomized controlled trial. Intervention and control wards were defined based on geofenced delivery of social media advertisements, not random assignment. This clarification has been added to the Service Delivery Data sections (“Administrative data on the number of HPV vaccinations provided during the July 16–20 MNCH week were obtained from local government health authorities. In two area councils—the Abuja Municipal Area Council (AMAC, population 4.2 million) and Kuje Area Council (population 527,473)—wards were classified as intervention or control areas based on whether geofenced social media advertisements promoting HPV vaccination were delivered to caregivers residing in those wards. Assignment of wards to intervention or control status was based on programmatic considerations rather than randomization.”)

(c) We clarify that not all caregivers in intervention wards necessarily received or recalled messages, and that ward-level analyses assume partial exposure (“Not all caregivers residing in intervention wards were necessarily exposed to or recalled the digital messages. Ward-level comparisons therefore reflect differences in vaccination uptake in areas targeted by the digital campaign, rather than individual-level exposure effects.”)

(d–e) We provide additional detail on how eligibility was determined, how systematic sampling was implemented, and how field supervisors monitored adherence (“Within each data collection site, interviewers used systematic sampling, approaching every second eligible caregiver. Eligibility was determined by confirming that the respondent was a caregiver of a girl aged 9–17 years who had attended the facility or pharmacy during the MNCH week. Field supervisors monitored interviewer adherence to sampling procedures through spot checks and real-time review of submitted survey forms.”)

(f–g) In study limitations, we acknowledge potential selection and response biases associated with recruitment at pharmacies and the absence of response-rate data, and we discuss how these may affect interpretation (“Finally, because participants were recruited at health facilities and pharmacies, the sample may over-represent caregivers who are more health-engaged, potentially limiting generalizability.”).

(h) We have added a new table, Table 3, summarizing service delivery statistics for intervention and control wards to improve transparency and provided a clearer explanation of the findings. (“Table 3. Shows estimated additional HPV vaccinations delivered during the July 16–20 MNCH week, by intervention status and area council. Administrative data showed that 6,516 HPV vaccinations were administered in the Abuja Municipal Area Council (AMAC) during the July 16–20 MNCH week, corresponding to vaccination of approximately 11.9% of the estimated population of girls aged 9–14 (54,831). This figure reflects overall service delivery during the recovery period rather than the effect of the digital intervention. In ward-level regression analyses adjusting for ward population (not shown), intervention wards in AMAC delivered an estimated 185 more vaccinations per ward than control wards. Multiplying this per-ward difference by the nine intervention wards yields an estimated 1,665 additional vaccinations attributable to the campaign, corresponding to an approximately 3.0-percentage-point increase relative to the council-level denominator (54,831 girls aged 9–14). In the Kuje area council, administrative data indicated that 984 HPV vaccinations were administered during the July 16–20 MNCH week, corresponding to vaccination of approximately 14.3% of the estimated population of girls aged 9–14 (6,857). As in AMAC, this figure reflects overall service delivery during the recovery period rather than the effect of the digital intervention. In ward-level regression analyses adjusting for ward population (not shown), intervention wards delivered an estimated 37 more vaccinations per ward than control wards. Multiplying this per-ward difference by the six intervention wards yields an estimated 222 additional vaccinations attributable to the campaign, corresponding to an approximately 3.2-percentage-point increase relative to the council-level denominator (6,857 girls aged 9–14).”)

Major Comment 4

“More limitations should be included…”

Response:

We thank the reviewer for these suggestions and have expanded the Limitations section to explicitly address:

• Potential selection bias related to pharmacy-based recruitment and systematic sampling

• Possible response bias

• Potential spillover or cross-fertilization between intervention and control wards

• The likely direction of recall and social desirability bias, which may overestimate the strength of associations

(“ The study’s strengths include the use of both caregiver exit survey data and administrative service delivery statistics, which provided convergent evidence for the observed associations. However, several limitations should be noted. Data on both adolescent vaccination status and exposure to the social media campaign were caregiver-reported, introducing the potential for response bias, including recall and social desirability biases, which may have led to overestimation of the strength of observed associations. In addition, the cross-sectional study design precludes causal inference.

Although an instrumental variable analysis presented no evidence that unobserved factors correlated with Facebook use were driving the observed association between message recall and vaccination status, the exclusion restriction was imperfect. Frequency of Facebook use may be associated with vaccination behavior through pathways other than message recall, such as broader health information seeking or engagement with health services. As such, the instrumental variable findings should be interpreted cautiously and as supportive rather than definitive.

Finally, because participants were recruited at health facilities and pharmacies using systematic sampling, the sample may over-represent caregivers who are more health-engaged, potentially limiting generalizability. In addition, spillover or cross-fertilization of messages between intervention and control wards—through interpersonal communication or exposure to shared media environments—may have attenuated observed differences between study groups.”

Minor Comments

1. Scope beyond service disruption:

We clarify that the intervention was designed for a period of service disruption, while noting its potential relevance for broader HPV vaccination efforts (“Although the intervention was designed to support HPV vaccine uptake during a temporary service disruption, the findings may also inform demand-generation strategies for routine HPV vaccination when services are available more consistently.”).

2. Mechanism of action:

We now explicitly state that messages helped caregivers know when and where vaccination services were available, in addition to increasing motivation (“In addition to increasing motivation, the messages provided practical information about when and where HPV vaccination services were available during MNCH week, helping caregivers translate intent into action.”).

3. Message content:

We expanded the manuscript to include a clearer description of the content and engagement strategies used in the social media advertisements. Specifically, we describe how messages addressed common caregiver concerns, featured trusted messengers such as pharmacists, emphasized the limited availability of services during MNCH week, and encouraged caregivers to sign up to receive notifications when vaccination services became available. This description is now included in the Introduction as part of the campaign description. (“Message content and engagement strategy. The digital market priming campaign employed multiple short-form social media advertisements informed by prior behavioral insights research and iterative testing. Advertisements featured locally relevant themes and trusted messengers, including community pharmacists, and were designed to address common caregiver concerns about HPV vaccination, such as safety, cost, eligibility, and fertility-related misconceptions. In response to service disruptions and uncertainty about vaccine availability, a subset of advertisements encouraged caregivers to sign up to receive notifications when HPV vaccination services became available in their area. This approach allowed the campaign to maintain engagement during periods of service interruption and enabled follow-up messaging when vaccination opportunities arose. Messages consistently emphasized that HPV vaccination was free, time-limited, and available for girls aged 9–14 years, and provided information on where services could be accessed during MNCH week. Multiple creatives were rotated and adapted over time to improve relevance and engagement while maintaining consistent core themes related to motivation, trust, and caregivers’ ability to act. Caregiver recall of these messages was later assessed in the exit survey and used as the primary exposure variable in individual-level analyses.")

4. Instagram use:

We clarify that Facebook and Instagram ads were delivered jointly through Meta’s advertising platform and that platform-specific exposure was not measured separately (“Advertisements were delivered jointly across Facebook and Instagram using Meta’s advertising platform; platform-specific exposure could not be distinguished and was therefore not measured separately.”)

5. Caregiver-reported vaccination status:

We have made the suggested change in the Conclusion. (“A caregiver’s recall of vaccine messages on Facebook was strongly associated with caregiver-reported vaccination status.”)

REVIEWER #2

We thank Reviewer #2 for their positive assessment and helpful suggestions.

Vaccination status: We now more explicitly acknowledge the limitations of caregiver-reported vaccination status and clarify that no verification via vaccination cards or facility records was attempted (“Data on both adolescent vaccination status and exposure to the social media campaign were caregiver-reported, introducing the potential for response bias, including recall and social desirability biases, which may have led to overestimation of the strength of observed associations. In addition, the cross-sectional study design precludes causal inference.”)

• Digital reach and representativeness: That the intervention relies on Facebook and Instagram may exclude caregivers without access to these platforms has been addressed. (“As the intervention relied on digital advertising platforms, caregivers without regular internet or social media access may have been less likely to b

Attachment Submitted filename: 2025 12 28 Response to Reviewers.docx

# Editor report: Decision Letter 1

**DOI:** 10.1371/journal.pone.0352986.r003

**Authors:** Ameer Muhammad, Ameer Muhammad

6 Jan 2026

PONE-D-25-50622R1

Leveraging Social Media to Mitigate HPV Vaccine Service Disruptions in Abuja, Nigeria

PLOS One

Dear Dr. Agha,

Thank you for submitting your manuscript to PLOS ONE. After careful consideration, we feel that it has merit but does not fully meet PLOS ONE’s publication criteria as it currently stands. Therefore, we invite you to submit a revised version of the manuscript that addresses the points raised during the review process.

The study is well-executed and demonstrates clear improvement from the previous version. The authors have thoughtfully addressed prior reviewer comments, resulting in a stronger and scientifically balanced manuscript overall.

Abstract

• The period of the health worker strike lacks a specific year, which is necessary to contextualize the timing of disruption and distinguish it from other program phases.

• The description of “post-test-only exit survey” may unintentionally suggest an experimental structure; clarity is needed that this was an observational, cross-sectional design to avoid implying causality.

• The reference to the two-stage residual inclusion model would benefit from brief justification of its purpose, indicating it was used to assess potential bias from unobserved confounding rather than as a causal estimator.

• The reported 23.3 and 7.9 percentage-point increases are presented without explanation of derivation; clarification whether these are absolute differences or model-predicted marginal effects would improve scientific transparency.

• The statement that administrative data “corroborated” survey findings could be interpreted as causal validation; rephrasing to reflect consistency between data sources would maintain analytical neutrality.

• The concluding claim that the strategy “may strengthen HPV vaccine introduction and program resilience” extends beyond the evidence presented; it should reflect association rather than program-level impact.

Introduction

•  The introduction does not explicitly state the study objective or hypothesis at the end. It should close with a single sentence summarizing what the study aimed to test or examine (e.g., “This study assessed whether exposure to a social media priming campaign was associated with increased HPV vaccine uptake during MNCH week in Abuja.”).

•  The section on “5–10 percentage point behavior change” might inadvertently sound like a benchmark or expected outcome. It would strengthen rigor to clarify that these are contextual estimates from prior studies, not anticipated effects in this research.

•  The discussion of digital reach and social media campaign delivery metrics in the latter half (advertisement creatives, weekly reach, etc.) belongs in Methods rather than Introduction. Retaining only conceptual rationale for using digital platforms would keep focus on research framing rather than implementation detail.

•  The introduction assumes readers understand “market priming strategies” without defining it operationally for this context. A concise definition specific to this intervention (how priming was applied digitally to HPV vaccination) would add clarity.

•  The link between the Fogg Behavior Model and the actual study variables could be articulated more precisely — for example, by briefly explaining how “motivation,” “ability,” and “prompt” relate to message recall or vaccination behavior measured later.

•  The reference to behavioral insights-based messaging is accurate but might benefit from specifying whether any message pretesting or audience segmentation was conducted; this would ground the behavioral science claim more firmly.

•  The temporal alignment between the health worker strike, campaign implementation, and MNCH week should include exact dates or months to strengthen reproducibility.

Methodology

•  The study design description correctly identifies the assessment as combining survey and administrative data but does not clarify whether the data linkage was purely ecological or if any identifiers (e.g., facility, date) were matched. Clarifying this distinction would help readers understand the analytical unit.

•  The sampling approach (systematic, every second eligible caregiver) is reasonable; however, there is no mention of how sample size (271 caregivers) was determined. A brief note on sample size justification or power consideration would improve transparency.

•  The inclusion of private pharmacies introduces heterogeneity in the sampling frame. It would be scientifically useful to indicate whether analyses adjusted for type of recruitment site, or whether site differences were checked for bias (e.g., different exposure likelihoods between PHCs and pharmacies).

•  The data collection period lists dates as “16-7-2025 to 20-7-2025”; this narrow window might limit recall reliability for message exposure. It would strengthen interpretation to acknowledge or test for recall bias given proximity to campaign activity.

•  The ethical approval information is clear; however, the protocol number date combination (July 15, 2024, but “17/08/2024”) appears inconsistent. Verify and align dates for compliance accuracy.

•  The measurement of exposure (recall of HPV vaccine messaging) and instrument (Facebook use frequency) are appropriate but conceptually close. There is potential concern that Facebook use frequency may not meet the exclusion restriction required for a valid instrument, since heavier users could also differ in health-seeking behavior. A brief note in the results or discussion clarifying instrument validity tests would be important.

•  The statistical description is adequate but lacks mention of whether multicollinearity among control variables or goodness-of-fit of logistic models were assessed. Including this check would strengthen methodological rigor.

•  In the instrumental variable explanation, it would help to specify that the 2SRI approach was used rather than two-stage least squares because of the nonlinear outcome (binary vaccination variable). This contextualizes the analytic choice.

•  The ward-level analysis section appropriately describes ecological comparison but could add a statement clarifying whether any baseline vaccination data were available to assess pre-campaign differences between intervention and control wards. Without this, causal interpretation is limited.

•  The definition of intervention vs control wards relies on programmatic assignment without randomization. This should be clearly acknowledged as a non-random quasi-experimental comparison, with possible confounding from unobserved local factors.

•  The population denominators are estimated using projections and UN data, which is acceptable but introduces potential error. A brief acknowledgment of uncertainty in denominator estimates would demonstrate statistical transparency.

•  There is no mention of missing data handling (e.g., skipped questions, incomplete interviews). Even if none occurred, stating this explicitly (“No missing data were observed”) would confirm dataset completeness.

•  The software and version (Stata 14) is listed correctly, but the section does not specify significance thresholds (e.g., α = 0.05) or whether analyses were two-tailed, which are standard reporting expectations.

Results

The descriptive statistics in Sample Characteristics align internally; proportions sum correctly and correspond to the reported total (271). There is internal coherence between the narrative and Table 1, though the table presentation could be clarified: column headings (“P value (Column 3) n (%) P value”) appear duplicated and may obscure which variable corresponds to which p-value. Ensuring consistent column labeling (e.g., separate p-values for message recall and vaccination status) would improve interpretability.

The reported Facebook use frequencies and message recall rates are consistent across text and table. However, the proportion recalling HPV messaging (33.6%) seems high relative to population-level expectations; acknowledging potential exposure misclassification or recall bias would strengthen interpretation.

The bivariate associations correctly summarize statistical patterns from Table 1. The reported differences (43% vs 30.8% vs <20%) match the data, and the education–vaccination gradient (37.5% → 77.1%) is accurate. The description is sound and consistent with table values.

In Table 2, odds ratios and confidence intervals are internally coherent with corresponding p-values. There are no numerical inconsistencies between text and table for the key estimates (Facebook multiple times/day → AOR = 7.59 [3.55–16.19]; message recall → AOR = 3.36 [2.75–4.11]; higher education → AOR = 5.53 [1.45–21.08]). However, the table includes very wide confidence intervals for some categories (e.g., 40–49 years AOR = 0.73 [0.08–6.43]), indicating sparse data in certain strata. Mentioning this limitation would improve scientific transparency.

The transformation of odds ratios to “23.3 percentage-point difference” is reasonable if based on predicted probabilities, but the text should specify that these were marginal effects derived from logistic predictions rather than direct proportion differences to ensure clarity of interpretation.

The McFadden pseudo-R² values (6.75% and 11.56%) are low but typical for behavioral logistic models; noting that model fit is modest but acceptable would show analytical awareness.

The instrumental variable analysis appears correctly described. The non-significant residual (p = 0.736) supports absence of detectable endogeneity, but this test does not confirm the instrument’s validity. A single line clarifying that the test does not rule out all unobserved confounding would prevent overinterpretation.

In the service delivery analysis, figures for AMAC (6,516 vaccinations; 11.9% coverage of 54,831) and Kuje (984; 14.3% of 6,857) are mathematically consistent. The extrapolation from per-ward differences (185 × 9 = 1,665 and 37 × 6 = 222) is correct and yields the stated ~3 percentage-point increases. However, these estimates implicitly assume uniform denominators across wards and no pre-existing coverage differences. This assumption should be acknowledged to maintain analytic rigor.

The phrase “estimated additional vaccinations attributable to the campaign” remains causal in tone despite non-randomized allocation. It should be softened to “associated with intervention wards” to align with observational design.

Across the section, administrative coverage (≈ 12–14%) is substantially lower than survey-reported vaccination (64.2%). This discrepancy likely arises because survey respondents were caregivers already attending facilities. A short clarifying sentence would prevent reader confusion and reinforce that survey results reflect a service-user subset rather than population coverage.

Discussion

•  The summary accurately reflects findings, but the statement that administrative service delivery data corroborated these findings may overstate agreement. Recommend clarifying that only the direction of association, not magnitude, was consistent across data sources.

•  The large numerical difference between survey-reported vaccination (≈64%) and administrative coverage (≈12–14%) should be briefly acknowledged as reflecting different denominators (service users vs. population-level estimates).

•  The inference that digital priming “supported vaccine uptake” implies temporal causality. Given the cross-sectional design, clarify that this represents association, not confirmed causal effect.

•  The comparison with prior literature is well-supported, but the phrase “our observed effects fall squarely within this range” could be softened to “our results are comparable to prior findings” since the cited studies include quasi-experimental evidence.

•  The behavioral interpretation using the Fogg Behavior Model is logical but should specify that motivation and ability were inferred constructs, not directly measured variables.

•  The conclusion that “digital communication provides a buffer against service disruptions” is theoretically sound, but clarify that this is a conceptual inference, not empirically tested within the current dataset.

•  The finding that the effect of recall was independent of caregiver education is noteworthy; no modification needed here, this supports equitable reach.

•  The strengths and limitations section is balanced. The discussion of recall bias, social desirability bias, and cross-sectional design constraints is transparent and complete.

•  The note that the instrument (Facebook frequency) may not fully satisfy the exclusion restriction is excellent and shows methodological awareness, retain as is.

•  The limitation on digital access inequality is appropriate; it might be enhanced by adding that hybrid offline channels are necessary to mitigate structural inequities in access.

•  The observation that the sample may over-represent health-engaged caregivers should explicitly state how this could bias findings (likely upward, overestimating recall and vaccination rates).

•  The acknowledgment of spillover between intervention and control wards is strong. Suggest noting that this likely biased results toward the null, explaining modest observed differences.

•  The policy implications are relevant and practical; no factual issues detected. Adding a brief mention of evaluating equity outcomes alongside cost-effectiveness could enhance policy relevance.

•  In the Conclusion, replace the phrase “administrative data corroborated” with “administrative data were consistent with” to reflect the observational study design.

•  The statement that a “4-percentage-point increase could translate into thousands of additional girls protected” is persuasive but should include a qualifier — e.g., “if replicated at national scale.”

•  The closing argument that digital priming offers a “low-cost, scalable tool” is appropriate; no issue detected. The emphasis on future research into scalability and integration is well-placed.

Please submit your revised manuscript by Feb 20 2026 11:59PM. If you will need more time than this to complete your revisions, please reply to this message or contact the journal office at plosone@plos.org. When you're ready to submit your revision, log on to https://www.editorialmanager.com/pone/ and select the 'Submissions Needing Revision' folder to locate your manuscript file.

Please include the following items when submitting your revised manuscript:

- A letter that responds to each point raised by the academic editor and reviewer(s). You should upload this letter as a separate file labeled 'Response to Reviewers'.
- A marked-up copy of your manuscript that highlights changes made to the original version. You should upload this as a separate file labeled 'Revised Manuscript with Track Changes'.
- An unmarked version of your revised paper without tracked changes. You should upload this as a separate file labeled 'Manuscript'.

-->If you would like to make changes to your financial disclosure, please include your updated statement in your cover letter. Guidelines for resubmitting your figure files are available below the reviewer comments at the end of this letter.

If applicable, we recommend that you deposit your laboratory protocols in protocols.io to enhance the reproducibility of your results. Protocols.io assigns your protocol its own identifier (DOI) so that it can be cited independently in the future. For instructions see: https://journals.plos.org/plosone/s/submission-guidelines#loc-laboratory-protocols. Additionally, PLOS ONE offers an option for publishing peer-reviewed Lab Protocol articles, which describe protocols hosted on protocols.io. Read more information on sharing protocols at https://plos.org/protocols?utm_medium=editorial-email&utm_source=authorletters&utm_campaign=protocols.

We look forward to receiving your revised manuscript.

Kind regards,

Ameer Muhammad

Academic Editor

PLOS One

Journal Requirements:

If the reviewer comments include a recommendation to cite specific previously published works, please review and evaluate these publications to determine whether they are relevant and should be cited. There is no requirement to cite these works unless the editor has indicated otherwise.

Please review your reference list to ensure that it is complete and correct. If you have cited papers that have been retracted, please include the rationale for doing so in the manuscript text, or remove these references and replace them with relevant current references. Any changes to the reference list should be mentioned in the rebuttal letter that accompanies your revised manuscript. If you need to cite a retracted article, indicate the article’s retracted status in the References list and also include a citation and full reference for the retraction notice.

[Note: HTML markup is below. Please do not edit.]

[NOTE: If reviewer comments were submitted as an attachment file, they will be attached to this email and accessible via the submission site. Please log into your account, locate the manuscript record, and check for the action link "View Attachments". If this link does not appear, there are no attachment files.]

To ensure your figures meet our technical requirements, please review our figure guidelines: https://journals.plos.org/plosone/s/figures

You may also use PLOS’s free figure tool, NAAS, to help you prepare publication quality figures: https://journals.plos.org/plosone/s/figures#loc-tools-for-figure-preparation.

NAAS will assess whether your figures meet our technical requirements by comparing each figure against our figure specifications.

# Author comment: Author response to Decision Letter 2

**DOI:** 10.1371/journal.pone.0352986.r004

17 Feb 2026

Response to Reviewers

Manuscript ID: PONE-D-25-50622R1

Title: Leveraging Social Media to Mitigate HPV Vaccine Service Disruptions in Abuja, Nigeria

Dear Dr. Muhammad and Reviewers,

We thank the Academic Editor and reviewers for their careful review and constructive feedback. We appreciate the positive assessment of the study’s rigor and the clear guidance provided. We have revised the manuscript extensively to address all comments and to improve clarity, transparency, and alignment with the observational nature of the study. Below, we provide a detailed, point-by-point response indicating how each comment was addressed. Page and section references refer to the revised manuscript.

Abstract

Reviewer comment:

The period of the health worker strike lacks a specific year.

Response:

We have specified the year throughout the Abstract and manuscript. The Abstract now states that the strike occurred in 2025, with services restored during July 16–20, 2025 MNCH week .

Reviewer comment:

The phrase “post-test-only exit survey” may imply an experimental design.

Response:

We removed this phrasing and clarified that the study used a cross-sectional exit survey with an observational design. No causal language is used in the Abstract or elsewhere.

Reviewer comment:

Clarify the purpose of the two-stage residual inclusion (2SRI) model.

Response:

The Abstract now explicitly states that the 2SRI model was used as a sensitivity analysis to assess potential bias from unobserved confounding, not as a causal estimator.

Reviewer comment:

The reported percentage-point differences lack explanation.

Response:

We clarified that the 23.3 percentage-point estimate reflects model-predicted marginal effects, and that the 7.9 percentage-point difference represents an application of those marginal effects to the study population .

Reviewer comment:

“Administrative data corroborated” may imply causal validation.

Response:

We replaced this wording with “administrative data were consistent with survey findings” throughout the manuscript,

Reviewer comment:

The concluding claim overstates program-level impact.

Response:

We softened the conclusion to reflect association rather than impact, stating that digital priming may help maintain caregiver readiness rather than strengthen programs directly

Introduction

Reviewer comment:

The Introduction lacks a clear study objective.

Response:

We added a clear objective sentence at the end of the Introduction stating that the study assessed whether caregiver recall of a digital market priming campaign was associated with HPV vaccine uptake during a brief service resumption in Abuja, Nigeria.

Reviewer comment:

The discussion of “5–10 percentage point behavior change” could be misinterpreted as a benchmark.

Response:

We clarified that these figures are contextual estimates from prior studies, not expected or benchmark effects for this study.

Reviewer comment:

Campaign delivery metrics belong in Methods rather than Introduction.

Response:

All implementation details (reach, creatives, delivery) were moved to a dedicated “Message Content and Engagement Strategy” subsection in Methods. The Introduction now focuses on conceptual rationale only.

Reviewer comment:

Define “market priming strategies” operationally.

Response:

We added a concise operational definition tailored to HPV vaccination and service disruptions.

Reviewer comment:

Clarify how the Fogg Behavior Model relates to measured variables.

Response:

We now explicitly state that the model informed message design, while message recall and vaccination status are observable indicators, not direct measures of motivation or ability.

Reviewer comment:

Specify timing of strike, campaign, and MNCH week.

Response:

Exact months and dates are now provided consistently throughout the Introduction and Methods .

Methods

Reviewer comment:

Clarify whether survey and administrative data were linked.

Response:

We clarified that data linkage was ecological and ward-level, with no individual identifiers matched across data sources .

Reviewer comment:

Provide sample size justification.

Response:

We added a sample size statement indicating that 300 interviews were targeted, providing ±6 percentage-point precision accounting for clustering (design effect = 1.5), but that 271 interviews were achieved due to the short service window.

Reviewer comment:

Address heterogeneity introduced by private pharmacies.

Response:

We clarified the role of pharmacies, described their selection, and noted that analyses accounted for clustering by recruitment location.

Reviewer comment:

Acknowledge recall bias due to narrow data collection window.

Response:

Recall bias is now acknowledged explicitly in the Discussion, noting that proximity to campaign activity may inflate recall.

Reviewer comment:

Verify ethical approval dates.

Response:

Ethical approval information was reviewed and aligned. The protocol number and approval date now match the NHREC documentation.

Reviewer comment:

Facebook use frequency may violate the exclusion restriction.

Response:

We explicitly acknowledge this limitation, stating that the instrument may be correlated with health-seeking behavior, and that the 2SRI analysis should be interpreted as a sensitivity check only.

Reviewer comment:

Specify why 2SRI was used instead of 2SLS.

Response:

We clarified that 2SRI was used because the outcome variable is binary, making it appropriate for nonlinear models.

Reviewer comment:

Clarify non-random assignment of intervention wards.

Response:

We explicitly state that ward assignment was non-random and programmatic, and that ward-level analyses are quasi-experimental and observational.

Reviewer comment:

Acknowledge uncertainty in population denominators.

Response:

We added a statement noting uncertainty associated with population projections and UN-derived age proportions .

Reviewer comment:

Address missing data and model diagnostics.

Response:

We added a paragraph stating that no missing data were observed, that multicollinearity was assessed using variance inflation factors, and that standard goodness-of-fit statistics were used. Significance thresholds (α = 0.05, two-tailed tests) are now specified.

Results

Reviewer comment:

Table 1 column headings are unclear.

Response:

Table 1 headings were revised to clearly distinguish p-values for message recall and vaccination status.

Reviewer comment:

High recall rate may reflect misclassification or recall bias.

Response:

We explicitly acknowledge the possibility of recall bias and exposure misclassification arising from self-reported message recall in the Results and Discussion. We note that recall may reflect broader health engagement rather than verified exposure and interpret findings accordingly.

Reviewer comment:

Wide confidence intervals should be noted.

Response:

We added a sentence noting that wide confidence intervals reflect sparse data in certain strata.

Reviewer comment:

Clarify marginal effects interpretation.

Response:

We explicitly state that percentage-point differences are model-predicted marginal effects, not direct differences in observed proportions.

Reviewer comment:

“Attributable to the campaign” is causal in tone.

Response:

We replaced this phrasing with “associated with intervention wards” throughout the Results and Discussion.

Reviewer comment:

Explain discrepancy between survey and administrative coverage.

Response:

We added explicit clarification that survey vaccination reflects facility-attending caregivers and cumulative vaccination, whereas administrative data reflect population-level delivery.

Discussion and Conclusion

Reviewer comment:

Avoid causal language throughout.

Response:

We carefully reviewed and revised the Discussion and Conclusion to consistently use associational language, avoiding claims of causality or program-level impact.

Reviewer comment:

Clarify that administrative data agreement is directional, not magnitude-based.

Response:

We now state that administrative data were consistent in direction, not magnitude.

Reviewer comment:

Add qualifier to national-scale implications.

Response:

We added qualifiers such as “if replicated at larger scale” when discussing potential population impact .

Reviewer comment:

Strengthen equity discussion.

Response:

We explicitly note that the sample over-represents health-engaged caregivers and that digital strategies should be paired with offline approaches to mitigate access inequities.

We believe these revisions have strengthened the manuscript and aligned it fully with PLOS ONE’s standards for transparency and observational research. We thank the Editor and reviewers again for their valuable guidance and hope the revised manuscript is now suitable for publication.

Sincerely,

Sohail Agha, PhD

(on behalf of all authors)

Attachment Submitted filename: 2026 1 18 Response to Reviewers.docx

# Editor report: Decision Letter 2

**DOI:** 10.1371/journal.pone.0352986.r005

**Authors:** Ameer Muhammad, Ameer Muhammad, Ameer Muhammad

3 Jun 2026

PONE-D-25-50622R2

Leveraging Social Media to Mitigate HPV Vaccine Service Disruptions in Abuja, Nigeria

PLOS One

Dear Dr. Agha,

Thank you for submitting your manuscript to PLOS ONE. After careful consideration, we feel that it has merit but does not fully meet PLOS ONE’s publication criteria as it currently stands. Therefore, we invite you to submit a revised version of the manuscript that addresses the points raised during the review process.

==============================

Dear Dr. Agha and colleagues,

Thank you for the revised manuscript. You have addressed the main points from the previous round. The text now uses associational language throughout, the 2SRI model is presented as a sensitivity analysis rather than a causal test, and you have added the sample size justification, the note on non-random ward assignment, the explanation of the marginal effects, the model diagnostics, and the recall bias and equity limitations. I am satisfied with these changes.

I have one point to confirm and two smaller items to raise.

1. The NHREC protocol number does not match across the submission. The Ethics Statement gives NHREC/01/01/2007-15/07/2024, approved 15 July 2024, while the Methods section gives NHREC/01/01/2007-17/08/2024 with no date. Please check both against the approval letter and make the form and the manuscript agree.
2. The abstract stored in the submission system is still the earlier version. For example, it reads 33.8%, "assesses the effect," "to test endogeneity," and "corroborated," while the abstract in the manuscript file is the revised one. Production uses the manuscript abstract, but you may want to update the system field so the two match.
3. The confidence interval for the recall and vaccination association in Table 2 (AOR 3.36, 95% CI 2.75 to 4.11) is narrower than I would expect given the sample size and the other estimates in the model. Please confirm the model and the interval. If it is correct as reported, leave it as is.

Once the protocol details are confirmed, I expect to be able to accept the manuscript. Thank you.

==============================

Please submit your revised manuscript by Jul 18 2026 11:59PM. If you will need more time than this to complete your revisions, please reply to this message or contact the journal office at plosone@plos.org. When you're ready to submit your revision, log on to https://www.editorialmanager.com/pone/ and select the 'Submissions Needing Revision' folder to locate your manuscript file.

Please include the following items when submitting your revised manuscript:

- A letter that responds to each point raised by the academic editor and reviewer(s). You should upload this letter as a separate file labeled 'Response to Reviewers'.
- A marked-up copy of your manuscript that highlights changes made to the original version. You should upload this as a separate file labeled 'Revised Manuscript with Track Changes'.
- An unmarked version of your revised paper without tracked changes. You should upload this as a separate file labeled 'Manuscript'.

If you would like to make changes to your financial disclosure, please include your updated statement in your cover letter. Guidelines for resubmitting your figure files are available below the reviewer comments at the end of this letter.

If applicable, we recommend that you deposit your laboratory protocols in protocols.io to enhance the reproducibility of your results. Protocols.io assigns your protocol its own identifier (DOI) so that it can be cited independently in the future. For instructions see: https://journals.plos.org/plosone/s/submission-guidelines#loc-laboratory-protocols. Additionally, PLOS ONE offers an option for publishing peer-reviewed Lab Protocol articles, which describe protocols hosted on protocols.io. Read more information on sharing protocols at https://plos.org/protocols?utm_medium=editorial-email&utm_source=authorletters&utm_campaign=protocols.

As the corresponding author, your ORCID iD is verified in the submission system and will appear in the published article. PLOS supports the use of ORCID, and we encourage all coauthors to register for an ORCID iD and use it as well. Please encourage your coauthors to verify their ORCID iD within the submission system before final acceptance, as unverified ORCID iDs will not appear in the published article. *Only*  the individual author can complete the verification step; PLOS staff *cannot*  verify ORCID iDs on behalf of authors.

We look forward to receiving your revised manuscript.

Kind regards,

Ameer Muhammad

Academic Editor

PLOS One

Journal Requirements:

If the reviewer comments include a recommendation to cite specific previously published works, please review and evaluate these publications to determine whether they are relevant and should be cited. There is no requirement to cite these works unless the editor has indicated otherwise.

Please review your reference list to ensure that it is complete and correct. If you have cited papers that have been retracted, please include the rationale for doing so in the manuscript text, or remove these references and replace them with relevant current references. Any changes to the reference list should be mentioned in the rebuttal letter that accompanies your revised manuscript. If you need to cite a retracted article, indicate the article’s retracted status in the References list and also include a citation and full reference for the retraction notice.

[Note: HTML markup is below. Please do not edit.]

[NOTE: If reviewer comments were submitted as an attachment file, they will be attached to this email and accessible via the submission site. Please log into your account, locate the manuscript record, and check for the action link "View Attachments". If this link does not appear, there are no attachment files.]

To ensure your figures meet our technical requirements, please review our figure guidelines: https://journals.plos.org/plosone/s/figures

You may also use PLOS’s free figure tool, NAAS, to help you prepare publication quality figures: https://journals.plos.org/plosone/s/figures#loc-tools-for-figure-preparation.

NAAS will assess whether your figures meet our technical requirements by comparing each figure against our figure specifications.

# Author comment: Author response to Decision Letter 3

**DOI:** 10.1371/journal.pone.0352986.r006

12 Jun 2026

Response to Reviewers

Manuscript PONE-D-25-50622R2

Leveraging social media to mitigate HPV vaccine service disruptions in Abuja, Nigeria

Dear Dr. Muhammad,

Thank you for your positive assessment of our revised manuscript and for the opportunity to address the remaining points. We are grateful for your careful reading. Below we respond to each item in turn. Changes to the manuscript are shown using tracked changes in the revised file.

1. NHREC protocol number consistency

Editor comment:

The NHREC protocol number does not match across the submission. The Ethics Statement gives NHREC/01/01/2007-15/07/2024, approved 15 July 2024, while the Methods section gives NHREC/01/01/2007-17/08/2024 with no date. Please check both against the approval letter and make the form and the manuscript agree.

Response:

We thank the editor for catching this discrepancy. We checked both entries against the NHREC approval letter. The letter specifies two distinct identifiers: a Protocol Number (NHREC/01/01/2007-17/08/2024, corresponding to the date of receipt of the valid application) and an Approval Number (NHREC/01/01/2007-29/08/2024, corresponding to the date of final determination). The approval was granted on 29 August 2024 and is valid through 28 August 2025. The number previously stored in the submission system Ethics Statement field (NHREC/01/01/2007-15/07/2024, 15 July 2024) was a transcription error and does not appear in the approval letter.

To resolve the inconsistency, we have revised the Ethical Considerations section of the manuscript to state both identifiers and the approval and validity dates explicitly. The Methods section now reads:

The study protocol, including the consent process, was reviewed and approved by the National Health Research Ethics Committee of Nigeria (NHREC) (protocol number NHREC/01/01/2007-17/08/2024; approval number NHREC/01/01/2007-29/08/2024), approved on 29 August 2024 and valid through 28 August 2025 [16].

We have also corrected the Ethics Statement field in the submission system to match this text, so that the form and the manuscript now agree and both correspond to the approval letter.

2. Abstract stored in the submission system

Editor comment:

The abstract stored in the submission system is still the earlier version. For example, it reads 33.8%, “assesses the effect,” “to test endogeneity,” and “corroborated,” while the abstract in the manuscript file is the revised one. Production uses the manuscript abstract, but you may want to update the system field so the two match.

Response:

Thank you. We have replaced the abstract stored in the submission system with the current revised abstract from the manuscript file, so that the two versions now match. The system field no longer contains the earlier wording (e.g., “33.8%,” “assesses the effect,” “to test endogeneity,” or “corroborated”). No change to the manuscript was required for this item, as the manuscript already contained the revised abstract.

3. Confidence interval in Table 2 (recall and vaccination)

Editor comment:

The confidence interval for the recall and vaccination association in Table 2 (AOR 3.36, 95% CI 2.75 to 4.11) is narrower than I would expect given the sample size and the other estimates in the model. Please confirm the model and the interval. If it is correct as reported, leave it as is.

Response:

We re-estimated the model and confirm the coefficient and interval are correct as reported. The relatively narrow interval reflects the strong and precisely estimated association between recall and vaccination (z = 11.82), in contrast to the wider intervals on the sparser sociodemographic categories. We have left the estimate unchanged.

We believe these revisions fully address the remaining points. Thank you again for your time and for the constructive review of our manuscript.

Sincerely,

Sohail Agha, on behalf of all co-authors

Attachment Submitted filename: 2026 6 12 Response_to_Reviewers_R3.docx

# Editor report: Decision Letter 3

**DOI:** 10.1371/journal.pone.0352986.r007

**Authors:** Ameer Muhammad, Ameer Muhammad, Ameer Muhammad, Ameer Muhammad

18 Jun 2026

Leveraging Social Media to Mitigate HPV Vaccine Service Disruptions in Abuja, Nigeria

PONE-D-25-50622R3

Dear Dr. Agha,

We’re pleased to inform you that your manuscript has been judged scientifically suitable for publication and will be formally accepted for publication once it meets all outstanding technical requirements.

Within one week, you’ll receive an e-mail detailing the required amendments. When these have been addressed, you’ll receive a formal acceptance letter and your manuscript will be scheduled for publication.

An invoice will be generated when your article is formally accepted. Please note, if your institution has a publishing partnership with PLOS and your article meets the relevant criteria, all or part of your publication costs will be covered. Please make sure your user information is up-to-date by logging into Editorial Manager at Editorial Manager® and clicking the ‘Update My Information' link at the top of the page. For questions related to billing, please contact billing support.

If your institution or institutions have a press office, please notify them about your upcoming paper to help maximize its impact. If they’ll be preparing press materials, please inform our press team as soon as possible -- no later than 48 hours after receiving the formal acceptance. Your manuscript will remain under strict press embargo until 2 pm Eastern Time on the date of publication. For more information, please contact onepress@plos.org.

Kind regards,

Ameer Muhammad

Academic Editor

PLOS One

# Editor report: Acceptance letter

**DOI:** 10.1371/journal.pone.0352986.r008

**Authors:** Ameer Muhammad, Ameer Muhammad, Ameer Muhammad, Ameer Muhammad

PONE-D-25-50622R3

PLOS One

Dear Dr. Agha,

I'm pleased to inform you that your manuscript has been deemed suitable for publication in PLOS One. Congratulations! Your manuscript is now being handed over to our production team.

At this stage, our production department will prepare your paper for publication. This includes ensuring the following:

* All references, tables, and figures are properly cited

* All relevant supporting information is included in the manuscript submission,

* There are no issues that prevent the paper from being properly typeset

You will receive further instructions from the production team, including instructions on how to review your proof when it is ready. Please keep in mind that we are working through a large volume of accepted articles, so please give us a few days to review your paper and let you know the next and final steps.

Lastly, if your institution or institutions have a press office, please let them know about your upcoming paper now to help maximize its impact. If they'll be preparing press materials, please inform our press team within the next 48 hours. Your manuscript will remain under strict press embargo until 2 pm Eastern Time on the date of publication. For more information, please contact onepress@plos.org.

You will receive an invoice from PLOS for your publication fee after your manuscript has reached the completed accept phase. If you receive an email requesting payment before acceptance or for any other service, this may be a phishing scheme. Learn how to identify phishing emails and protect your accounts at https://explore.plos.org/phishing.

If we can help with anything else, please email us at customercare@plos.org.

Thank you for submitting your work to PLOS ONE and supporting open access.

Kind regards,

PLOS ONE Editorial Office Staff

on behalf of

Dr. Ameer Muhammad

Academic Editor

PLOS One
